# Efficacy of a hybrid psychoeducational and skills-based therapy (Trauma PORTAL) for adults with PTSD related to childhood interpersonal trauma: a parallel-group, randomised controlled trial

**DOI:** 10.1016/j.eclinm.2026.104003

**Published:** 2026-06-05

**Authors:** Dana C. Ross, Negar Sayrafizadeh, Louloua Ashikhusein Waliji, Kaniz Farhat, Annie K. Truuvert, Mahum Musheer, Simone N. Vigod, Cherry Chu, Sophie Soklaridis, David Rojas, Nancy McCallum

**Affiliations:** a Department of Psychiatry, Temerty Faculty of Medicine, University of Toronto, Toronto, Ontario, Canada; b Women's College Hospital, Toronto, Ontario, Canada; c Women's College Hospital and Research Institute, Toronto, Ontario, Canada; d Department of Obstetrics and Gynaecology, Temerty Faculty of Medicine, University of Toronto, Toronto, Ontario, Canada; e Centre for Addictions and Mental Health, Toronto, Ontario, Canada; f The Wilson Centre, University of Toronto/University Health Network, Toronto, Ontario, Canada

**Keywords:** Digital mental health, Psychoeducation, eHealth, Adverse childhood experiences, Group psychotherapy, Posttraumatic stress disorder

## Abstract

**Background:**

Many adults with posttraumatic stress disorder (PTSD) related to childhood interpersonal trauma (CIT) face substantial barriers to care and limited access to trauma-specific treatment. We evaluated the efficacy of the Trauma PORTAL (Providing Online tRauma Therapy using an Asynchronous Learning platform), a trauma-focused hybrid therapy integrating self-paced psychoeducational and skills-based modules and virtual therapist-led group sessions, to reduce PTSD symptoms in adults with a history of CIT.

**Methods:**

This randomised, assessor-masked, controlled, parallel-group trial was conducted at a single site in Ontario, Canada. Participants (≥18 years) with a history of CIT were recruited within an ambulatory urban hospital and met criteria for PTSD based on the Mini-International Neuropsychiatric Interview (MINI). Participants were randomly assigned (1:1) to Trauma PORTAL (intervention; eight online modules and eight optional weekly 1-h virtual group sessions facilitated by two trauma therapists) or treatment as usual (control). The primary outcome was severity of PTSD symptoms assessed at 8 weeks relative to baseline, measured by self-report on the PTSD Checklist for DSM-5 (PCL-5). Outcomes were analysed in the intention-to-treat population using a linear mixed-effects model. Secondary outcomes included clinician-rated PTSD severity using the Clinician-Administered PTSD Scale for DSM-5 (CAPS-5), emotion regulation, depression, anxiety, stress, and self-compassion. Individuals with lived experience of CIT were involved in the development of the Trauma PORTAL intervention, but not in the design of this trial. The trial was registered with ClinicalTrials.gov (NCT05670405).

**Findings:**

Between November 7, 2022, and October 6, 2023, 328 participants were screened for eligibility, and of these 183 (56%) provided consent and were enrolled. After 2 were lost to follow-up, 181 participants were randomly assigned to Trauma PORTAL (91 [50%]) or control (90 [50%]). At baseline, 147 (81%) participants were women, mean age was 40.8 years (SD 11.5), and 119 (66%) were White. Trauma PORTAL was superior to control in reducing PTSD symptoms, showing an adjusted mean difference (aMD) on the PCL-5 at week 8 of −7.08 (95% CI −11.55 to −2.61), corresponding to a moderate effect size (d = 0.44 [95% CI 0.12–0.76]). This effect was maintained at week 16 (aMD −7.00, 95% CI −11.83 to −2.18). No adverse events were reported.

**Interpretation:**

Trauma PORTAL reduced PTSD symptoms more than treatment as usual, supporting its potential as an effective and acceptable hybrid psychoeducation and skills-based therapy that may help expand access to trauma-focused care for adults with CIT. Further evaluation against established treatments is warranted.

**Funding:**

This project was supported by the WCHAMSG (Women's College Hospital Alternative Medical Staff Group) Innovation Fund of the Alternative Funding Plan for the Academic Health Sciences Centres of Ontario, and the Department of Psychiatry, Women's College Hospital.

**Research in context**

**Evidence before this study**

Adults with a history of childhood interpersonal trauma (CIT) are at elevated risk for posttraumatic stress disorder (PTSD) and other adverse mental and physical health outcomes. Guideline-recommended psychological treatments for PTSD are effective, but access remains limited across many care settings. Treatment interventions for adults with CIT that are both effective and scalable are needed. Psychoeducation and skills-based interventions represent a potential approach and are already embedded within several evidence-based PTSD treatments, yet have been less rigorously evaluated as primary treatments for PTSD, particularly among adults with CIT. We searched PubMed and the Cochrane Central Register of Controlled Trials up to Jan 31, 2022, using search terms “adverse childhood experiences,” “childhood interpersonal trauma,” “complex trauma,” “hybrid,” “blended,” “therapist-supported,” “psychoeducation,” and “PTSD,” restricted to English-language publications. We identified no randomised controlled trials evaluating a hybrid psychoeducational and skills-based group intervention designed as a primary treatment for PTSD in adults with CIT.

**Added value of this study**

To our knowledge, this is the first randomised controlled trial to evaluate a trauma-focused hybrid eight-week therapy combining self-paced online psychoeducational and skills-based modules with optional therapist-led virtual group sessions for adults with CIT. The study was conducted in a clinical care setting and evaluates whether a scalable, digitally delivered foundational trauma intervention can reduce PTSD symptoms compared to care-as-usual. In addition, the study examines key implementation outcomes, including recruitment, retention, adherence, and acceptability. By focussing on these objectives, the study seeks to contribute to the existing evidence on trauma care and highlight the potential role of these approaches in expanding access to PTSD treatment for adults with CIT. This work addresses a critical gap in access to trauma-focused care and evaluates whether lower-intensity, technology-enabled interventions can expand capacity while maintaining clinical benefit.

**Implications of all the available evidence**

Findings from this study suggest that psychoeducational and skills-based approaches, delivered in a structured hybrid format, can support meaningful symptom improvement among adults with CIT. These findings extend a limited evidence base for psychoeducation-focused interventions as primary treatments for PTSD and indicate that clinically relevant change is possible using hybrid models. Further research is needed to compare such interventions directly with established treatments and to clarify their effectiveness across different clinical settings and service contexts.

## Introduction

Childhood interpersonal trauma (CIT) refers to experiences of abuse, neglect, or other forms of harm occurring within caregiving or relational contexts. CIT is a widespread public health concern associated with increased risk of mental illness, chronic disease, and early mortality.^1^ Despite growing recognition of its broad and enduring impacts across the lifespan, most trauma-affected adults face significant barriers to accessing psychological treatment. Front-line mental health staff are often tasked with supporting adults with complex trauma histories within brief, group-based, or multidisciplinary service models. The resource demands associated with training and supervising clinicians in evidence-based trauma-focused modalities contribute to limited service availability and long waitlists, particularly within publicly funded or under-resourced systems.^2^ As a result, developing treatment approaches that are both feasible and adaptable to diverse care contexts is an increasing priority for mental health service delivery.^3^

Digital interventions are emerging as promising strategies to improve the availability of psychological treatments and have demonstrated effectiveness across common mental health conditions, including Posttraumatic Stress Disorder (PTSD), depression, and anxiety.^4^ Evidence suggests that digital interventions tend to be most effective when they include some degree of therapist support or guidance.^5^ Hybrid formats that combine self-paced digital components with therapist support offer greater flexibility in delivery and the potential to extend therapist capacity. In practice, this structure may support broader integration into routine care settings while preserving access to therapeutic support. Evidence from such approaches, including trauma-focused cognitive behavioural therapy (TF-CBT) integrating digital self-help with brief clinician contact, suggests feasibility and meaningful symptom improvement.^6^^,^^7^ Evaluating whether such hybrid approaches can achieve reductions in PTSD symptoms among adults with histories of CIT is an important next step.

Psychoeducation and skills-based content are core components of several evidence-based trauma-focused treatments, including Cognitive Processing Therapy (CPT) and TF-CBT. Within these approaches, psychoeducation is used to support understanding of symptoms and to promote engagement with treatment. More broadly, psychoeducation has been associated with improvements in insight, self-efficacy, and adaptive coping across clinical populations.^8^ In a study of a web-based program for complex dissociative disorders, both clients and therapists described engagement with psychoeducational materials as fostering understanding and a sense of safety, alongside perceived gains in insight and self-compassion.^9^ When delivered in a structured and standardised format, psychoeducation may be particularly well suited to hybrid delivery models, offering a pragmatic approach to targeting PTSD symptoms while maintaining consistency across participants. Psychoeducation is most often delivered as part of a broader, multi-component trauma interventions, rather than as a stand-alone treatment for PTSD. Related interventions, including phase-based skills-focused approaches such as Skills Training in Affective and Interpersonal Regulation (STAIR), have demonstrated efficacy in improving PTSD symptoms and related domains such as emotional regulation and interpersonal functioning, including in trauma-exposed populations.^10^ However, the effectiveness of psychoeducation and skills-based interventions for reducing PTSD symptoms among adults with histories of CIT has not been well established.

Most guideline-recommended trauma therapies are designed for individual delivery, reflecting a stronger and more consistent evidence based for individual trauma-focused treatments, which can be resource-intensive and challenging to implement broadly across mental health services.^11^ Group-based interventions have demonstrated meaningful benefits for PTSD^12^, and when combined with incorporating psychoeducation and skill-building, group formats may extend the reach of evidence-based approaches while supporting relational engagement and shared learning. Despite this potential, relatively few studies have evaluated group-based interventions for adults with a history of CIT, and evidence on their acceptability, safety, and mechanisms of change remains limited.^13^

To address these gaps, we developed the Trauma PORTAL (Providing Online tRauma Therapy using an Asynchronous Learning platform), a trauma-focused hybrid psychoeducational and skills-based therapy designed to expand availability of trauma care for adults with CIT, with an optional therapist-led weekly group component intended to provide additional support without limiting accessibility. It combines self-paced online modules with therapist-led virtual 1-h group sessions, offering a structured, lower-resource approach to symptom reduction. The primary outcome was severity of PTSD symptoms assessed at 8 weeks relative to baseline, measured by self-report on the PTSD Checklist for DSM-5 (PCL-5). Secondary outcomes included PCL-5 change at week 16, and clinician-rated PTSD severity using the CAPS-5, emotion regulation, depression, anxiety, stress, and self-compassion. If effective, the Trauma PORTAL may offer a feasible approach to delivering trauma-focused care within existing mental health services.

## Methods

### Study design and participants

We conducted a parallel-group, randomised, assessor-masked, controlled trial at an ambulatory university teaching hospital in Toronto, Canada. This trial compared a hybrid, eight-week psychoeducation and skill-based intervention (Trauma PORTAL) with usual care, defined as remaining on the waitlist for the hospital-based Trauma Therapy Program (TTP) with access to synchronous, group-based therapy following the study period. Outcomes were measured at baseline, 8 weeks, and at 16 weeks, with 8 weeks being the timepoint at which the primary PTSD symptom outcome, utilising the PCL-5, was evaluated. Participants were recruited from a mandatory virtual orientation session for the hospital's TTP which provides treatment for adults with a history of CIT.

Participants were recruited from the TTP waitlist, where referral and acceptance into the program are based on a history of childhood interpersonal trauma. As such, all participants had a pre-established history of childhood interpersonal trauma prior to study enrolment. Eligible participants were adults (≥18 years) with a PTSD diagnosis, established using the Mini-International Neuropsychiatric Interview (MINI, Module H,^14^, based on qualifying Criterion A traumatic event, which could occur at any point in the lifespan, and self-reported CIT (physical, sexual, emotional abuse, or neglect) before age 18, internet access, and English proficiency. Exclusion criteria were active/unstable substance use disorder within the past three months, active mania, psychosis, or current suicidal ideation, psychiatric hospitalisation within the past six months, or prior participation in a TTP group.

All candidates completed a clinical assessment by a TTP trauma therapist to confirm eligibility and rule out safety risks, significant cognitive impairments, or substantial self-regulation or case management needs. Participants receiving concurrent mental health care elsewhere (psychological or psychopharmacological) were not excluded. Written informed consent was obtained before baseline assessments. We involved people with relevant lived experience throughout the initial development and evaluation of the Trauma PORTAL which have been previously published.^15^

### Ethics

The study was approved by the Women's College Hospital Research Ethics Board (REB #2022-0083-E) and registered with ClinicalTrials.gov (NCT05670405).

### Randomisation and masking

Participants were randomly assigned in a 1:1 ratio to the Trauma PORTAL or a treatment as usual group. Because the study was conducted within a hospital-based trauma therapy program with long wait times, this control design reflected the usual care context. Participants were recruited in four cohorts of about 25 to accommodate scheduling and group delivery. Randomisation was conducted by the study coordinator using a computer-generated sequence within Research Electronic Data Capture (REDCap;^16^, after baseline assessments were completed, without stratification. Allocation was concealed from participants until after assignment.

Outcome assessors (trained research assistants) were masked to group allocation throughout data collection. All assessors completed the National Center for PTSD Clinician-Administered PTSD Scale for DSM-5 Training Curriculum (both the didactic as well as the interviews with virtual simulated patients) before administering interviews to ensure standardised rating procedures. Upon completion of the training, assessors completed mock interviews with the study Principal Investigator to ensure consistency in administration and scoring prior to conducting study assessments. If an assessor was inadvertently unblinded, such as through participant disclosure, the participant's file was reassigned to a different masked assessor. Assessors were only informed of group allocation after the final follow-up or participant withdrawal. Therapists and participants were not masked to allocation due to the nature of the intervention. Statistical analyses were conducted with treatment groups coded as A and B to maintain blinding during analysis.

### Procedures

The TTP has offered a synchronous (in-person or virtual) eight-week psychoeducational and skill-building group for adults with a history of CIT for nearly two decades. Building on the content from this group, our research team developed a hybrid version, the Trauma PORTAL, comprising (1) eight asynchronous online modules and (2) eight optional, therapist-led weekly 1-h virtual groups. Participants were granted access to all Trauma PORTAL modules upon enrolment and could complete the content at their own pace over an 8-week period. While the intervention was designed to be completed over approximately 8 weeks (one module per week), modules were not restricted or sequentially released. Participants’ access to the modules expired following week 8 of the intervention and were encouraged to complete at least 75% of the modules within this timeframe to be considered as having meaningfully engaged with the intervention.

In addition to the asynchronous modules, participants were offered optional weekly 60-min therapist-led virtual group sessions that took place concurrently during the intervention period. These semi-structured sessions provided an opportunity for participants to discuss module content, ask the two group co-facilitators questions, practise new skills, and receive relational support in a structured therapeutic setting, while facilitators reviewed key concepts from the modules and supported application of skills, rather than delivering fully manualized or fixed session content. The group facilitators were trauma therapists working in the TTP who consented to participate in the study and were familiar with the content of the online modules, as they had offered the synchronous version as part of regular TTP programming previously. To maximise accessibility, group attendance was optional, allowing participation by individuals with caregiving responsibilities, variable work or school schedules, or health-related limitations that could make fixed weekly sessions difficult to attend.

The Trauma PORTAL modules incorporated psychoeducation, cognitive and emotion regulation skills, and guided practice exercises designed to enhance self-awareness, grounding, and relational safety. Each module followed a consistent structure, video or text-based teaching, reflection prompts, and brief experiential activities, taking approximately 45–60 min to complete. Module content is outlined in Appendix p 2, and the development of the Trauma PORTAL^15^ and the results of the pilot study^17^ are reported elsewhere.

Assessments occurred at baseline, week 8 (post-intervention), and week 16 (follow-up), and included online self-report questionnaires and structured telephone interviews for rater-reported outcomes. Adverse events were monitored via optional weekly surveys. After completing the Trauma PORTAL, intervention group participants were invited to a 20-min virtual exit interview as part of standard clinical care. These interviews informed post-intervention treatment planning but were not part of the study protocol and no additional intervention-specific treatment was introduced in our program to participants during this period between week 8 and week 16. Post-intervention disposition was documented through chart review; control participants did not complete exit interviews and remained on the waitlist.

### Outcomes

Assessments occurred at baseline, 8 weeks (end of the intervention), and 16 weeks after randomisation. The primary outcome was PTSD symptom severity on the self-reported PCL-5 at 8 weeks. Secondary outcomes included PCL-5 symptom severity at week 16, as well as a further measure of PTSD symptom severity to assist with comparison to other studies: the CAPS-5.^18^ The CAPS-5, a structured clinical interview, was conducted by trained research staff to evaluate current symptom severity and diagnostic status. Higher scores indicate greater PTSD symptom severity.

Additional secondary outcomes, assessed at baseline and at weeks 8 and 16, included the Depression Anxiety Stress Scales–21 (DASS-21), a brief self-report instrument that evaluates depression, anxiety, and stress over the past week across three subscales.^19^ Higher scores indicate greater symptom severity across the subscales. Emotion regulation was assessed using the Difficulties in Emotion Regulation Scale–18 (DERS-18), which captures deficits across six domains such as emotional awareness, clarity, and impulse control.^20^ Total scores are calculated by summing item responses, with higher scores indicating greater difficulties in emotion regulation. Self-compassion was measured using the 12-item Self-Compassion Scale–Short Form (SCS-SF), which assesses key domains including self-kindness, mindfulness, and common humanity.^21^ The total score is calculated as the mean of all items after reverse scoring negative subscales. Childhood adversity was assessed using the 10-item Adverse Childhood Experiences (ACE) questionnaire.^22^ Items are scored dichotomously (0 = no, 1 = yes) and summed to yield a total score ranging from 0 to 10, with higher scores indicating greater exposure to adverse childhood experiences.

We also evaluated process outcomes to inform future implementation and scalability, including recruitment metrics (e.g., number screened, enrolled, and eligible), retention rates across timepoints, adherence (completion of modules and optional groups), and acceptability (an acceptability questionnaire along with weekly feedback forms for patient participants and semi-structured interviews with group facilitators). Safety was monitored throughout the 8-week intervention period via weekly self-report check-ins and ongoing communication with group facilitators and research staff.

The research team created three brief study-specific questionnaires to gather participant feedback and track practical aspects of implementation. These included the weekly Iterative Feedback Form (IFF; Appendix p 3), used to collect participants' comments about any technical or usability issues with the PORTAL website for ongoing review by the research team; the Health Services Utilisation Questionnaire (HSUQ), which recorded participants' use of health and related services at baseline and at the 8-week timepoint; and the post-intervention Intervention Acceptability Questionnaire (IAQ), which asked about participants' perceptions of the intervention's relevance, feasibility, and overall acceptability. Digital readiness was assessed with the General Internet Attitude Scale (GIAS;^23^; Appendix pp 4–5). Items for the GIAS and IAQ were rated on a Likert-type scale. Responses were analysed with counts and proportions calculated for each response category.

To evaluate implementation and participant experience, we measured adherence through clinical chart review, defined as completion of at least 75% of Trauma PORTAL modules, and attendance at optional weekly virtual groups. Finally, group facilitators completed a 30-min post-intervention debrief interview (Appendix p 6) to reflect on the intervention and share implementation challenges; these sessions were audio-recorded and transcribed.

### Choice of a primary measure

The PCL-5 was selected *a priori* as the primary outcome for its strong psychometric properties, clinical relevance, and suitability for remote delivery.^24^ It is a widely used self-report of PTSD symptom severity aligned with DSM-5 criteria, with strong internal consistency, test–retest reliability and demonstrated sensitivity to change in treatment trials. The brevity and self-administered format of the PCL-5 support high completion rates in longitudinal and digitally delivered interventions, particularly when compared with clinician-administered assessments such as the CAPS-5, which can be more time-intensive and burdensome for participants.

The PCL-5 is a 20-item self-report measure with total scores ranging from 0 to 80, with higher scores indicating greater symptom severity. A score of ≥31 was used to indicate probable PTSD, consistent with recommended cut-offs for clinical and research samples. Prior work indicates that a ≥10-point reduction is an indicator of treatment response (National Center for PTSD).

### Statistical analysis

Power calculations were based on detecting a between-group difference in change in PTSD symptom severity between groups on the PCL-5, as defined above. Assuming a two-sided α = 0.05, 80% power, and up to 20% attrition, a total sample of 200 participants (100 per arm) was targeted to detect a between-group difference of approximately 7 points. Using a standard deviation (SD) of 14.0 points based on baseline PCL-5 scores in the study sample, this corresponds to a moderate standardised effect size (Cohen's d ≈ 0.50). Participants were enrolled in four cohorts of about 50 individuals (∼25 per arm) to maintain balanced allocation and operational feasibility. The primary analysis was conducted using a linear mixed-effects model to account for repeated measures and within-subject correlation over time. Accordingly, the sample size calculation should be interpreted as an approximate planning estimate based on the expected between-group difference at the primary endpoint, rather than a model-based power calculation for the final mixed-effects model.

All analyses followed CONSORT guidelines. After trial commencement, the statistical analysis plan was updated to specify a linear mixed-effects model for repeated measures as the primary analytic approach, rather than ANCOVA, to account for within-subject correlations over time. ANCOVA was retained as a sensitivity analysis. The primary outcome and assessment timepoints were unchanged. This amendment was approved by the Research Ethics Board.

The primary outcome was analysed using a linear mixed-effects model with restricted maximum likelihood (REML), consistent with best practice for repeated measures data. PTSD symptom severity was modelled with fixed effects for visit (a categorical variable with the timepoints: baseline, week 8, week 16), group, and the visit-by-group interaction, a participant-level random intercept, and the best-fitting covariance structure for each model. The primary endpoint was the adjusted mean difference (aMD) derived from the visit-by-group contrast, between groups at week 8 and week 16 on the PCL-5 using separate models for each endpoint. As a sensitivity analysis, a complete-case ANCOVA was fitted at week 8 with baseline PCL-5 as a covariate. Although baseline scores were adjusted for as a covariate in the ANCOVA sensitivity analyses, they were included as part of the repeated measures outcome in the main mixed models to allow for proper modelling of the full trajectory. Effect sizes were calculated using unadjusted group means and pooled standard deviations. Missingness in the primary analyses was limited to repeated outcome measurements; predictors and covariates included in the models were complete.

Responder status, defined as a ≥10-point reduction on the PCL-5 from baseline, was analysed using logistic regression with group as the main predictor and baseline PCL-5 as a covariate, with missing outcomes imputed using multiple imputation by chained equations (MICE) under a missing-at-random (MAR) assumption. Logistic regression analyses were conducted separately within each imputed dataset and estimates pooled using Rubin's rules to provide odds ratios with 95% confidence intervals (CIs). The MAR assumption was further tested by modelling the association between missingness and observed data such as group and baseline score. No significant associations were reported, suggesting that MAR is a valid assumption. Observed complete-case proportions are also reported descriptively using chi-square tests. In a pre-specified subgroup analysis, responder status was also examined among participants with clinically elevated baseline symptoms (PCL-5 ≥31) only. All analyses were conducted under the intention-to-treat (ITT) principle, with significance defined as two-sided p < 0.05 and 95% CIs.

Secondary outcomes (CAPS-5, DASS-21, DERS-18, SCS-SF) were analysed using the same mixed model framework, with sensitivity analyses conducted using complete-case ANCOVA while adjusting for baseline score. Baseline demographic and clinical characteristics were comparable between groups, supporting the assumption of covariate balance through randomisation. Analyses of secondary outcomes were not adjusted for multiple comparisons and should be interpreted as exploratory. All analyses were conducted using SAS (version 9.4). The funders had no role in study design, data collection, data analysis, data interpretation, or writing of the report. The corresponding author had full access to all data in the study and had final responsibility for the decision to submit for publication.

### Role of the funding source

The funders of the study were not involved in designing the study, recruitment procedures, data collection, data analysis, data interpretation, writing of reports, or the decision to submit the manuscript for publication.

## Results

**Fig. 1:**
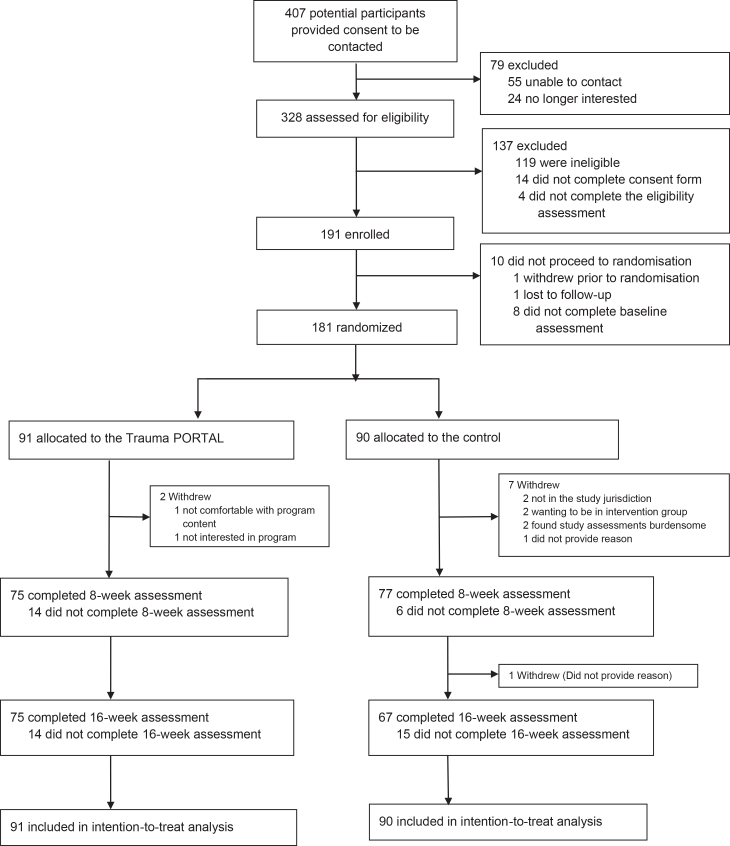
CONSORT flow diagram. ITT = intention-to-treat. PORTAL = Providing Online Trauma Therapy using an Asynchronous Learning platform.

Between Nov 7, 2022, and Oct 6, 2023, 407 individuals provided consent to be contacted by the study team (Fig. 1). Fifty-five did not respond to follow-up, and 24 declined participation. Of 328 individuals assessed for eligibility, 191 met inclusion criteria after completing two eligibility assessments, of which one withdrew prior to randomisation, one was lost to follow-up, and 8 did not complete the baseline assessment. A total of 181 participants were randomly assigned to the Trauma PORTAL group (n = 91) or control (n = 90).

Reasons for ineligibility (n = 119) included no PTSD diagnosis (n = 62), recent substance use (n = 24), recent psychiatric hospitalisation (n = 7), active suicidal ideation (n = 5), current manic symptoms (n = 5), current psychotic symptom (n = 3), limited English proficiency (n = 1), and other reasons (n = 12). The recruitment rate was 55% (181 of 328 screened), and retention at the primary outcome timepoint (week 8) was 84% (152 of 181 randomised). The final follow-up assessment was completed on Jan 22, 2024.

Baseline demographic and clinical characteristics are presented in Table 1. At baseline, 48 (53%) participants in each group reported concurrent engagement in non–trauma-specific mental health services, with patterns of health service utilisation similar between groups at baseline and remaining comparable across follow-up timepoints (Appendix p 7). Participants reported mean ACE scores of 5.6 (SD 2.1) in the Trauma PORTAL group and 5.6 (SD 2.2) in the control group (Appendix p 8). ACE category distributions did not differ between groups (Table 2).

**Table 1:** Baseline demographic characteristics of randomised study participants (N = 181). Characteristics are shown as N (%) unless otherwise specified.

Demographics/Categories	PORTAL (n = 91)	Control (n = 90)
Age in year (SD)	39.8 (11.3)	41.7 (11.7)
Biological sex		
Male	11 (12%)	8 (9%)
Female	78 (86%)	81 (90%)
Intersex	0 (0)	0 (0)
Prefer not to say	2 (2%)	0 (0)
Gender		
Man	10 (11%)	8 (9%)
Woman	75 (82%)	72 (80%)
Two-spirit	0 (0)	<5 (<5%)
Non-binary	<5 (<5%)	6 (7%)
Self-identify	0 (0)	<5 (<5%)
Prefer not to say	<5 (<5%)	<5 (<5%)
Education		
Elementary/some secondary	4 (4%)	0 (0)
Completed secondary school	6 (7%)	4 (4%)
Some university or college	20 (22%)	21 (23%)
Completed university or college	60 (66%)	64 (71%)
Prefer not to say	0 (0)	0 (0)
Employment		
Full-time	34 (37%)	38 (42%)
Part-time	9 (10%)	7 (8%)
Casual	6 (7%)	<5 (<5%)
Not currently employed	33 (36%)	35 (39%)
Prefer not to say	8 (9%)	<5 (<5%)
Annual income ($)		
>80,000	17 (19%)	24 (27%)
40,000–80,000	27 (30%)	25 (28%)
20,000–40,000	9 (10%)	12 (13%)
<20,000	18 (20%)	15 (17%)
Prefer not to say	19 (21%)	13 (14%)
Languages spoken at home		
English	85 (93%)	84 (93%)
French	6 (7%)	6 (7%)
Other	32 (35%)	34 (38%)
Ethnicity		
White or European	59 (65%)	60 (67%)
Black or Afro-Caribbean	10 (11%)	6 (7%)
Latino or Hispanic	8 (9%)	8 (9%)
East Asian or Asian	9 (10%)	<5 (<5%)
South Asian or Indian	7 (8%)	5 (6%)
Middle Eastern or Arab	<5 (<5%)	0 (0)
Indigenous (First Nations, Inuit, Métis)	<5 (<5%)	<5 (<5%)
Prefer not to say	<5 (<5%)	<5 (<5%)
Don't know	0 (0)	0 (0)
Other	5 (6%)	9 (10%)

Demographics Age presented as mean (SD); all other values are n (%).

**Table 2:** ACE category breakdown of those who completed the measure (n = 178). Characteristics are shown as n (%) unless otherwise specified.

ACE Type^a^	Category	PORTAL (n = 90)	Control (n = 88)
Household dysfunction	Substance abuse	45 (50.0)	45 (51.1)
	Parental separation/divorce	48 (53.3)	44 (50.0)
	Household member with mental Illness/suicide attempt	69 (76.7)	66 (75.0)
	Domestic violence towards mother/stepmother	34 (37.8)	36 (41.0)
	Incarcerated household member	12 (13.3)	16 (18.2)
Abuse	Psychological	81 (90.0)	80 (91.0)
	Physical	54 (60.0)	55 (62.5)
	Sexual	49 (54.4)	48 (54.6)
Neglect	Emotional	75 (83.3)	70 (79.6)
	Physical	30 (33.3)	35 (39.8)

ACE categories are based on the original CDC–Kaiser Permanente Adverse Childhood Experiences questionnaire.

a ACE = Adverse Childhood Experiences.

Module engagement among Trauma PORTAL participants (n = 91) was high, with participants completing a mean of 6.6 (SD 2.6) of eight self-paced modules (Appendix p 9). Seventy-two participants (79%) completed six or more modules, meeting the prespecified 75% module completion threshold. Attendance at the optional weekly therapist-led virtual group sessions was moderate. Trauma PORTAL participants attended a mean of 3.3 (SD 2.9) of eight sessions (Appendix p 9). Sixty-eight participants (75%) attended at least one group session, and 28 (31%) attended six or more.

Post-intervention clinical disposition was assessed through routine clinician-led exit interviews conducted as part of standard TTP procedures (Appendix p 10). No participants were assessed as not appropriate for further trauma-focused therapy at this time, and no adverse events or safety concerns were reported during the trial.

**Fig. 2:**
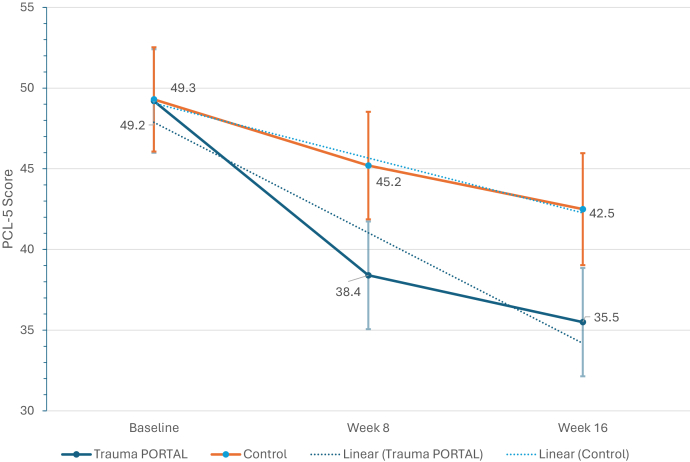
Adjusted mean PCL-5 scores over time by group. Points represent model-estimated adjusted means, with error bars indicating 95% confidence intervals, derived from intention-to-treat mixed-effects models.

The primary intention-to-treat analysis, including 181 randomised participants, showed that Trauma PORTAL was superior to control (Table 3). The adjusted mean difference on the PCL-5 at 8-weeks post-baseline was −7.08 (95% CI –11.55 to −2.61; p = 0.002), corresponding to a moderate effect size (Cohen's *d* = 0.44 [95% CI 0.12–0.76]; Fig. 2). Adjusted mean scores decreased from 49.2 (95% CI 46.2–52.2) to 38.2 (95% CI 35.0–41.4) in the PORTAL group, and from 49.3 (95% CI 46.3–52.4) to 45.3 (95% CI 42.1–48.4) in the control group. Twenty-eight participants (16%) had missing data for the primary outcome at week 8; no baseline variables predicted missingness, and sensitivity analyses using complete-case ANCOVA produced results consistent with the primary mixed-model analysis (Appendix p 11).

**Table 3:** Comparisons between Trauma PORTAL and control on the primary and secondary outcome measures (intention-to-treat analysis).

Outcome Measure	Timepoint	PORTAL unadjusted mean (SD); n	Control unadjusted mean (SD); n	Adjusted mean difference (95% CI)	p value	Cohen's d effect size (95% CI)
PTSD severity (PCL-5)	Baseline	49.18 (13.89); 90	49.44 (14.27); 88	–	–	–
	Week 8	38.76 (14.50); 76	45.39 (15.26); 78	−7.08 (−11.55 to −2.61)	0.002	0.44 (0.12–0.76)
	Week 16	36.12 (16.93); 77	43.03 (17.80); 69	−7.00 (−11.83 to −2.18)	0.005	0.40 (0.07–0.73)
PTSD severity (CAPS-5)	Baseline	34.48 (9.48); 91	34.06 (9.56); 90	–	–	–
	Week 8	27.23 (10.97); 70	29.54 (12.94); 69	−2.02 (−5.43 to 1.39)	0.244	0.19 (−0.14 to 0.53)
	Week 16	24.48 (12.28); 56	28.28 (13.82); 61	−3.19 (−7.01 to 0.63)	0.101	0.29 (−0.07 to 0.65)
Emotion Regulation (DERS)	Baseline	52.29 (13.09); 90	53.77 (13.17); 88	–	–	–
	Week 8	49.17 (13.17); 76	52.96 (13.34); 78	−4.36 (−8.37 to −0.35)	0.033	0.29 (−0.03 to 0.60)
	Week 16	47.40 (12.53); 77	53.40 (14.59); 69	−5.60 (−9.69 to −1.51)	0.008	0.44 (0.11–0.77)
Self-Compassion (SCS)	Baseline	2.34 (0.75); 90	2.22 (0.66); 88	–	–	–
	Week 8	2.65 (0.86); 76	2.43 (0.68); 78	0.29 (0.06–0.51)	0.013	0.29 (0.03–0.61)
	Week 16	2.78 (0.77); 77	2.48 (0.78); 68	0.30 (0.07–0.54)	0.010	0.38 (0.05–0.71)
Depression (DASS-21)	Baseline	22.82 (11.40); 90	22.71 (12.30); 88	–	–	–
	Week 8	19.00 (11.76); 76	22.23 (11.55); 78	−3.45 (−7.02 to 0.12)	0.058	0.28 (−0.04 to 0.59)
	Week 16	17.64 (11.90); 77	20.70 (13.02); 69	−2.09 (−5.78 to 1.60)	0.266	0.25 (−0.08 to 0.57)
Anxiety (DASS-21)	Baseline	17.09 (9.63); 90	17.91 (10.81); 88	–	–	–
	Week 8	15.63 (9.02); 76	18.33 (9.70); 78	−2.64 (−5.66 to 0.38)	0.086	0.29 (−0.03 to 0.61)
	Week 16	14.18 (9.77); 77	17.88 (11.21); 69	−3.13 (−6.24 to −0.01)	0.049	0.35 (0.03–0.68)
Stress (DASS-21)	Baseline	21.91 (8.24); 90	22.93 (9.47); 88	–	–	–
	Week 8	20.29 (9.44); 76	22.51 (9.53); 78	−2.39 (−5.20 to 0.42)	0.096	0.23 (−0.08 to 0.55)
	Week 16	19.40 (9.71); 77	21.97 (10.69); 69	−2.55 (−5.51 to 0.42)	0.092	0.25 (−0.07 to 0.58)

Data are mean (SD); n unless otherwise indicated. Baseline values are unadjusted descriptive statistics. Adjusted mean differences (95% CI) are reported for follow-up timepoints. Adjusted mean difference were estimated from intention-to-treat mixed-effects models comparing Trauma PORTAL with control.

PCL5 = PTSD Checklist for DSM-5. CAPS-5 = Clinician-Administered PTSD Scale for DSM-5. DERS = Difficulties in Emotion Regulation Scale. SCS-SF = Self-Compassion Scale-Short Form. DASS-21 = Depression, Anxiety and Stress Scale-21 Items.

At week 16, the between-group difference was maintained, with lower PCL-5 scores in the PORTAL group than in the control (aMD −7.00 [95% CI –11.83 to −2.18]; p = 0.005; *d* = 0.40 [95% CI 0.07–0.73]; Fig. 2). Adjusted mean scores decreased from 49.2 (95% CI 46.0–52.4) to 35.5 (95% CI 32.1–38.8) in the PORTAL group, and from 49.3 (95% CI 46.1–52.6) to 42.5 (95% CI 39.0–45.9) in the control group. Thirty-five (19%) of the 181 participants had missing data for the primary outcome at week 16.

At week 8, 52.6% of participants in the PORTAL group versus 28.6% in the control group achieved a ≥10-point reduction on the PCL-5 (Table 4). In the ITT logistic regression with multiple imputation adjusting for baseline PCL-5, the odds of response were significantly higher with PORTAL (OR 2.74 [95% CI 1.39–5.43]). At week 16, 62.3% of PORTAL participants versus 39.7% of controls achieved a ≥10-point reduction (OR 2.14 [95% CI 1.13–4.05]). Among participants with clinically elevated baseline symptoms (PCL-5 ≥31), PORTAL remained superior at both week 8 (OR 2.55 [95% CI 1.26–5.14]) and week 16 (OR 2.26 [95% CI 1.33–5.35]). Detailed responder outcomes are summarised in Table 4.

**Table 4:** Responder analysis on the PCL-5 by timepoint.

Outcome	PORTAL n/N (%)	Control n/N (%)	Absolute risk difference (95% CI)	Adjusted OR (95% CI)	p value
≥10-point reduction^a^ (week 8)	40/76 (52.6%)	22/77 (28.6%)	24.1% (9.0–39.2)	2.74 (1.39–5.43)	0.004
≥10-point reduction^a^ (week 16)	48/77 (62.3%)	27/68 (39.7%)	22.6% (6.7–38.5)	2.14 (1.13–4.05)	0.016
Clinically elevated baseline^b^ ≥10-point reduction (week 8)	38/70 (54.3%)	22/68 (32.4%)	21.9% (5.8–38.1)	2.55 (1.26–5.14)	0.009
Clinically elevated baseline^b^ ≥10-point reduction (week 16)	46/70 (65.7%)	26/62 (41.9%)	23.8% (7.2–40.3)	2.26 (1.33–5.35)	0.006

PCL-5 = PTSD Checklist for DSM-5. PORTAL = Trauma PORTAL hybrid psychoeducational intervention. Control = treatment as usual.

n/N (%) indicates the number of participants meeting response criteria divided by the number with available outcome data at that timepoint.

a Clinically significant improvement = ≥10-point reduction from baseline.

b Clinically elevated baseline subgroup = participants with baseline PCL-5 ≥31.

CAPS-5 were lower in the PORTAL group than in controls at both follow-up assessments, but between-group differences were not significant (week 8 *d* = 0.19 [95% CI –0.14 to 0.53]; week 16 *d* = 0.29 [95% CI –0.07 to 0.65]; Table 3). Sensitivity analyses at both timepoints showed consistent, non-significant effects favouring PORTAL. Detailed secondary analysis outcomes are summarised in Appendix p 13.

DERS-18 improved significantly more in the PORTAL group than in controls (week 8 *d* = 0.29 [95% CI 0.03–0.60]; week 16 *d* = 0.44 [95% CI 0.11–0.77]). SCS-SF also improved significantly at both timepoints (week 8 *d* = 0.29 [95% CI 0.03–0.61]; week 16 *d* = 0.38 [95% CI 0.05–0.71]; Table 3). Complete-case sensitivity analyses confirmed significant group differences for both measures at week 8, consistent with mixed-model results (Appendix p 12).

On the DASS-21, between-group differences were not significant for depression or stress. A marginally significant difference was observed for anxiety at week 16 (*p* = 0.049), favouring PORTAL (Table 3). Across subscales, effects were small to moderate (week 8 *d* = 0.29; week 16 *d* = 0.35), indicating modest overall symptom improvement. Sensitivity analyses showed significant differences for depression and anxiety at week 8, but not for stress, mirroring the direction of effects in the primary analysis.

Acceptability data were available for 76 participants (Appendix p 13). Most participants found the content helpful (92%) and easy to use (72%), and more than two-thirds (72%) strongly agreed they would recommend Trauma PORTAL to others with a history of childhood interpersonal trauma. The asynchronous format was highly valued, with 78% strongly agreeing that flexible, self-paced access was important. Completion of the participant weekly iterative feedback forms was low (Appendix p 14), and facilitator debriefs were limited, with three facilitators completing a total of six debriefs; responses were therefore summarised descriptively rather than formally analysed (Appendix p 15–16).

## Discussion

In the Trauma PORTAL study, we investigated the efficacy of a virtual trauma-focused hybrid psychoeducational and skills-based therapy for PTSD compared with treatment as usual. To our knowledge, this is the first randomised controlled trial to evaluate self-paced online modules combined with therapist-led virtual group sessions for adults with a history of CIT. The Trauma PORTAL produced a reduction in PTSD symptoms, corresponding to a moderate effect (*d* = 0.44) on the PCL-5 at eight weeks. Improvement was stable at week 16, and results were consistent across sensitivity analyses. Secondary outcomes showed significant improvements in emotion regulation and self-compassion, with a small but significant effect for anxiety at week 16, while depression, stress, and clinician-rated PTSD (CAPS-5) did not differ significantly between groups.

The moderate effect on self-reported PTSD symptoms observed in the Trauma PORTAL is consistent with findings from other digital trauma interventions. In the *Finding Solid Ground* RCT, an adjunctive online psychoeducational and skill-building program added to usual psychotherapy produced moderate between-group improvements in PTSD symptoms, emotion regulation, and self-compassion at six months.^25^ Meta-analytic evidence indicates that cognitive behavioural therapy (CBT)-based, internet-delivered interventions yield moderate effects (*d* = 0.61) compared with treatment as usual.^26^ Although effect sizes for traditional face-to-face therapies tend to be larger, hybrid or therapist-assisted digital formats achieve meaningful outcomes with reduced clinician time. Evidence from the low-intensity SPRING trial further supports that trauma-focused CBT can be effectively delivered in guided self-help formats with brief therapist contact.^7^ Taken together, these findings suggest that the contribution of the Trauma PORTAL lies in demonstrating that symptom reduction can be achieved within a lower-intensity, scalable model suited to routine care settings. Further research should compare hybrid trauma interventions with established trauma-focused therapies rather than waitlist or treatment-as-usual controls to clarify their relative effectiveness and guide implementation.

Although clinician-rated PTSD severity (CAPS-5) showed consistent effects favouring Trauma PORTAL at both follow-up timepoints, between-group differences were not statistically significant. Completion of the clinician-administered CAPS-5 was lower than for the self-reported PCL-5 (for example, 139 CAPS-5 interviews were completed at week 8 compared with 154 PCL-5 assessments), resulting in wider confidence intervals and reduced power to detect between-group differences. This may reflect the greater time and emotional burden associated with clinician-administered interviews compared with self-report measures, particularly in remote trials. In contrast, the PCL-5 was selected *a priori* as the primary outcome to maximise data completeness and minimise attrition bias, and demonstrated consistent improvements in self-reported PTSD symptoms favouring Trauma PORTAL. Together, these findings suggest that differences between self-reported and clinician-rated outcomes may reflect multiple factors, including differences in sensitivity to change, measurement context, and statistical power, rather than a single explanatory factor.

A treatment response was defined as a ≥10-point reduction on the PCL-5. In the Trauma PORTAL group, 53% achieved this threshold at week 8 and 60% at week 16, approximately twice that of controls. Comparable responder rates have been reported for individual therapist-assisted digital trauma therapies, including internet-delivered CBT for PTSD (71%;^27^, a telehealth program delivering CPT or Prolonged Exposure (PE) (65%;^28^, and an online prolonged-exposure trial (67%;^29^ The Trauma PORTAL model's group component allows greater reach than individual formats, supporting scalable access to measurable improvements in PTSD symptoms. The optional therapist-led group component was included to incorporate therapist support, which has demonstrated effectiveness, while maintaining broad accessibility through flexible participation.

All participants met diagnostic criteria for PTSD and reported high adverse childhood experience scores (mean 5.6) indicating substantial trauma exposure and comorbidity risk. Despite this high symptom burden, the Trauma PORTAL was well accepted and feasible to deliver within a routine hospital program. The module completion rate of 79% compares favourably with rates reported in digital therapist-assisted PTSD interventions, where completion typically ranges from 60% to 79%.^30^^,^^31^ The dropout rate (15%) matched pooled averages of 16% observed across psychological therapies for PTSD in general^32^ and was lower than the 36% dropout reported in trauma-focused guided self-help interventions.^33^ No adverse events were reported, consistent with evidence that well-structured and guided digital interventions for PTSD are well tolerated.

Evidence indicates that trauma-focused approaches incorporating psychoeducation and skills training can be effective for adults with complex trauma histories, although findings across studies remain mixed.^34^^,^^35^ Structured psychoeducation and skills training have been shown to reduce PTSD and dissociation symptoms and enhance emotion regulation in individuals with complex dissociative disorders.^36^ Building on this, recent work highlights how the capacity to take in and trust supportive information from others can also help people engage more fully in trauma treatment.^37^ Other studies show that individuals with complex trauma histories respond comparably to established trauma-focused treatments such as prolonged exposure, trauma-focused CBT, and eye movement desensitisation and reprocessing (EMDR).^38^^,^^39^ Clarifying which therapeutic elements most contribute to recovery, and how they can be delivered efficiently and equitably across diverse contexts, remains an important direction for future research.

This study has several strengths, including its randomised, assessor-masked design, high retention, and consistent findings across analytic approaches. This trial extends earlier work by demonstrating feasibility and efficacy in adults with CIT. The inclusion of both self-report and clinician-rated measures allowed a multidimensional evaluation of outcomes, and the high completion rate supports the intervention's acceptability and feasibility in routine clinical care.

Limitations include the absence of an active comparator, which restricts interpretation relative to established trauma-focused therapies, including synchronous group-based interventions delivered within the same clinical program, and the lack of long-term follow-up. Clinician-rated improvements did not reach statistical significance, which may reflect lower completion of clinician-administered assessments and differences between clinician-rated and self-report measures of change, an area for future investigation. Findings may not generalise to populations with limited digital access or in-patient settings. Because attendance at optional group sessions varied within the intervention arm, the study cannot determine the relative contribution of the self-paced modules versus the therapist-led groups; future trials should experimentally vary the level of therapist support to clarify these effects.

In summary, this randomised controlled trial provides evidence that a hybrid psychoeducational and skills-based therapy can significantly reduce PTSD symptoms in adults with histories of CIT. The Trauma PORTAL structure, combining self-paced modules with brief clinician contact, was acceptable, safe, and was associated with treatment response over the intervention period. These findings highlight the potential of hybrid trauma therapies that integrate psychoeducation, skill development, and relational support to expand access within systems where long wait times and limited clinician capacity remain major barriers. Future research should examine long-term outcomes, cost-effectiveness, and implementation at scale, and explore mechanisms of change to inform sustainable adoption.

## Contributors

DCR and NM were coprincipal investigators and wrote the funding application. Author DCR: conceptualisation, drafting the original proposal, methodology, funding acquisition, supervision, data analysis, writing—original draft, review, and editing. Author NS: data collection and analysis, managed the raw data, directly accessed and verified the underlying data, writing—original draft, review, and editing. Author LAW: conceptualisation, drafting the original proposal, data collection and analysis, writing—original draft, review, and editing. Author KF: data collection and analysis, writing—review, and editing. Author AKT: conceptualisation, drafting the original protocol, data collection and analysis, writing—review, and editing. Author MM: conceptualisation, data collection, writing—review, and editing. Author CC: was the project statistician, data analysis, drafting the statistical methods section, writing—review, and editing. Author SV: conceptualisation, methodology, writing—review, and editing. Author SS: conceptualisation, methodology, writing—review, and editing. Author DR: conceptualisation, methodology, writing—review, and editing. Author NM: conceptualisation, methodology, funding acquisition, data collection, supervision, writing—original draft, review, and editing. DCR, NS, and NM accessed and verified the data. All authors contributed and commented on the manuscript and approved the final version. All authors had access to all data in the study and had final responsibility for the decision to submit for publication.

## Data sharing statement

Protocols, consent forms, the statistical analysis plan, and deidentified quantitative data that underlie the results reported in this Article will be available upon reasonable request to the corresponding author, following institutional approval. Because the dataset includes sensitive information related to trauma history and mental health, public deposition was not appropriate.

## Declaration of interests

DCR is a member of the Scientific Committee for the International Society for the Study of Trauma and Dissociation. This is an unpaid position. DCR is a co-director of the Trauma Therapy Network of Ontario at Women's College Hospital in Ontario, Canada, and receives protected time for this role. NM is a co-director of the Trauma Therapy Network of Ontario at Women's College Hospital in Ontario, Canada and received protected time for this role. NM is the Program Lead of the Trauma Therapy Program (TTP) at Women's College Hospital in Ontario, Canada, and receives protected time for this role. All other authors declare no competing interests.

## Appendix A Supplementary data

Supplementary Tables

Protocol
