# Supplementary material for: Efficacy of a hybrid psychoeducational and skills-based therapy (Trauma PORTAL) for adults with PTSD related to childhood interpersonal trauma: a parallel-group, randomised controlled trial
Source: eClinicalMedicine. 2026 Jun 5;96:104003. doi: 10.1016/j.eclinm.2026.104003 (PMC13266226; doi:10.1016/j.eclinm.2026.104003)
Supplement: Supplementary Tables [file mmc1.docx]

**Table of Contents**

[Appendix Table 1: Trauma PORTAL Module Content 2](#_Toc228352961)

[Appendix Table 2. Iterative Feedback Form (IFF) 3](#_Toc228352962)

[Appendix Table 3. General Internet Attitudes Scale (GIAS) scores 4](#_Toc228352963)

[Appendix Table 4. Group Faciliator Debrief Questionnaire 6](#_Toc228352964)

[Appendix Table 5. Health Service Utilization Questionnaire (HSUQ) characteristics 7](#_Toc228352965)

[Appendix Table 6. ACE score breakdown 8](#_Toc228352966)

[Appendix Table 7. Online self-paced module completion 9](#_Toc228352967)

[Appendix Table 8. Attendance of Trauma PORTAL participants 9](#_Toc228352968)

[Appendix Table 9. Post-intervention clinical disposition of the Trauma PORTAL participants within the Trauma Therapy Program 10](#_Toc228352969)

[Appendix Table 10. Comparisons between Trauma PORTAL and control on the primary and secondary outcome measures (complete-case ANCOVA analysis) 11](#_Toc228352970)

[Appendix Table 11. Comparisons between Trauma PORTAL and control on the primary and secondary outcome measures (intention-to-treat, TimeXGroup ANCOVA analysis) 12](#_Toc228352971)

[Appendix Table 12. Sensitivity analyses for complete-case ANCOVA. 13](#_Toc228352972)

[Appendix Table 13. Participant responses to the Trauma PORTAL acceptability questionnaire 14](#_Toc228352973)

[Appendix Table 14. Responses to the optional Iterative Feedback Form (IFF) 15](#_Toc228352974)

[Appendix Table 15. Facilitator Debrief Findings 16](#_Toc228352975)

[Appendix Table 16. Missing Data by Outcome 18](#_Toc228352976)

## Appendix Table 1: Trauma PORTAL Module Content

|  | Topics/Skills/Movement Breaks |
| --- | --- |
| Module 1 | Psychoeducation: Introduction to Trauma Recovery  Skills: Self-Soothing Strategies; Breathing Exercises  Mindful Movement: Chair Sequence |
| Module 2 | Psychoeducation: The Neurobiology of Trauma  Skills: Mindful Stretching; Mindful noticing & Soothing; Self-nurturing  Mindful Movement: Focus on the Spine Movement |
| Module 3 | Psychoeducation: Emotional Regulation; The Survival Responses  Skills: What is Grounding?  Mindful Movement - Moving Your Joints |
| Module 4 | Psychoeducation: Managing Activation; What is Dissociation?  Skills: Tools for Identifying Activation and How to Respond; Body Scan Meditation  Mindful Movement: Moving on the Mat |
| Module 5 | Psychoeducation: Trauma-related Thinking Patterns: Intro to Cognitive Behavioural Therapy  Skills: Challenge Automatic Thoughts; Examine Cognitive Distortions; Box Breathing  Mindful Movement: Low Impact Fitness |
| Module 6 | Psychoeducation: Exploring Boundary Styles  Skills: Communication Strategies; Container Exercise  Mindful Movement: Boundary Exploration |
| Module 7 | Psychoeducation: Understanding and Responding to Reenactments; The Inner Critic  Skills: Personal Garden Visualization  Mindful Movement: Focusing on the Face |
| Module 8 | Psychoeducation: The Importance of Play, Creativity & Celebration  Skills: Goal Setting for Ongoing Healing  Mindful Movement: Seated Stretch |

## Appendix Table 2. Iterative Feedback Form (IFF)


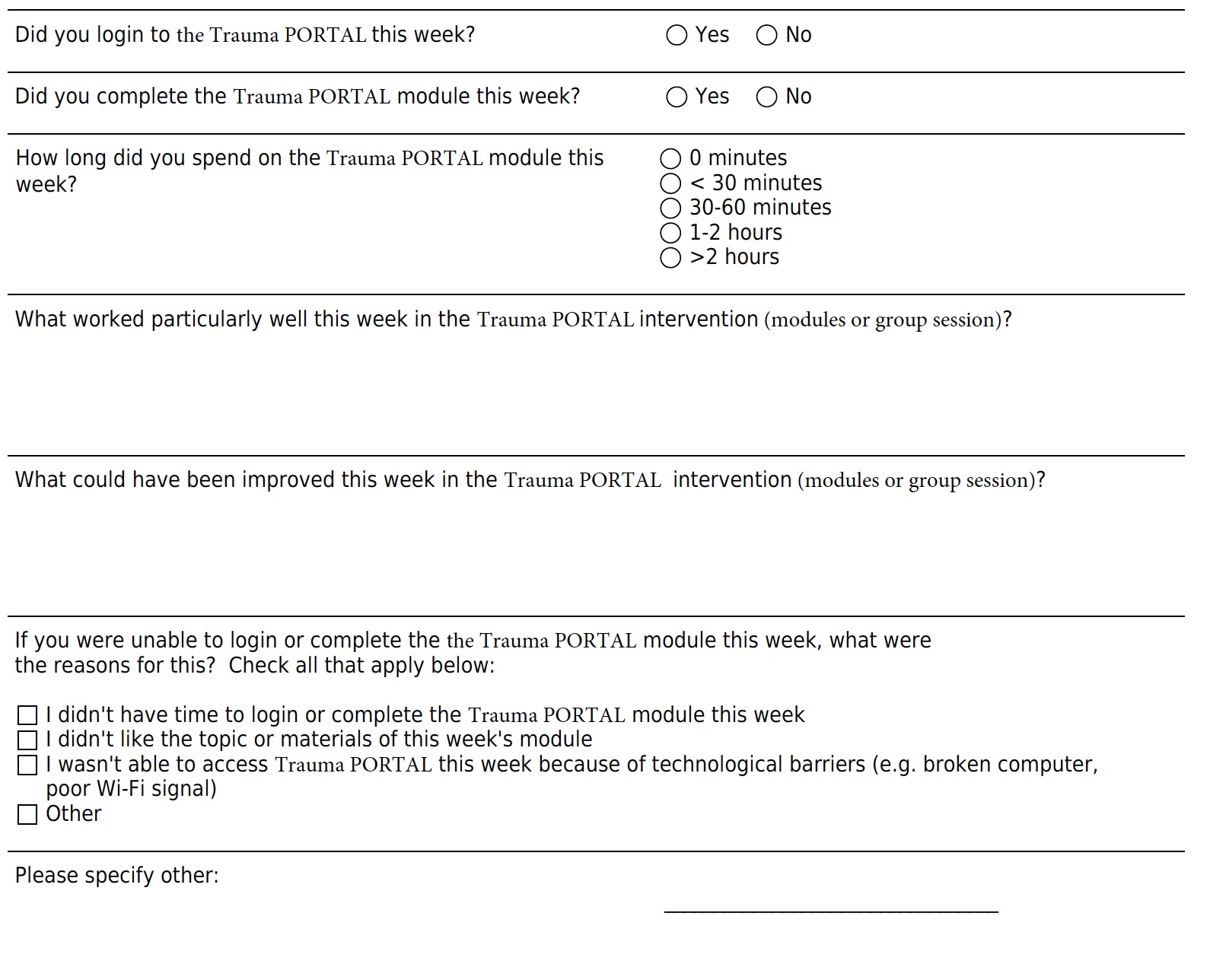


# **Appendix Table 3. General Internet Attitudes Scale (GIAS) scores** among completers: PORTAL (n = 90) and Control (n = 89).

| Statement | Strongly Disagree | | Slightly Disagree | | No Opinion | | Slightly Agree | | Agree | |
| --- | --- | --- | --- | --- | --- | --- | --- | --- | --- | --- |
|  | PORTAL | Control | PORTAL | Control | PORTAL | Control | PORTAL | Control | PORTAL | Control |
| The Internet makes a positive contribution towards society. | 1 | 2 | 13 | 11 | 11 | 7 | 33 | 37 | 32 | 32 |
| The Internet makes me feel anxious. | 16 | 14 | 16 | 20 | 16 | 10 | 32 | 32 | 10 | 13 |
| Using the Internet can cause health problems. | 4 | 11 | 16 | 11 | 17 | 17 | 35 | 32 | 18 | 17 |
| The Internet makes life more efficient. | 0 | 0 | 2 | 4 | 5 | 2 | 35 | 37 | 47 | 45 |
| I feel overwhelmed by the Internet. | 24 | 26 | 17 | 16 | 17 | 14 | 25 | 23 | 7 | 9 |
| The idea of going on the Internet gives me a thrill. | 20 | 18 | 22 | 15 | 27 | 38 | 20 | 15 | 1 | 2 |
| The Internet does not threaten me. | 6 | 4 | 13 | 15 | 24 | 17 | 17 | 21 | 30 | 31 |
| I would like to stay on the Internet for as long as I can. | 25 | 30 | 27 | 20 | 20 | 24 | 14 | 8 | 4 | 5 |
| The Internet makes me feel uncomfortable. | 28 | 26 | 27 | 20 | 16 | 22 | 16 | 15 | 3 | 5 |
| The use of the Internet is enhancing our standard of living. | 3 | 4 | 9 | 12 | 16 | 8 | 37 | 32 | 25 | 32 |
| The Internet makes me feel annoyed. | 14 | 16 | 17 | 13 | 21 | 15 | 31 | 34 | 7 | 9 |
| The Internet is dehumanising to society. | 12 | 15 | 21 | 11 | 22 | 22 | 27 | 30 | 6 | 10 |
| The Internet makes a great contribution to human life. | 1 | 1 | 8 | 11 | 14 | 13 | 37 | 37 | 30 | 26 |
| I feel bewildered by the Internet. | 33 | 28 | 22 | 16 | 22 | 26 | 12 | 13 | 1 | 5 |
| Using the Internet is harmful to people. | 10 | 10 | 22 | 20 | 24 | 18 | 34 | 36 | 0 | 4 |
| I feel intimidated by the Internet. | 35 | 41 | 23 | 13 | 12 | 24 | 17 | 9 | 2 | 1 |
| The thought of going on the Internet is exciting to me. | 17 | 17 | 14 | 19 | 32 | 31 | 24 | 15 | 3 | 5 |
| The Internet is bringing us into a bright new era. | 4 | 5 | 5 | 14 | 27 | 25 | 34 | 19 | 20 | 25 |
| I feel disheartened at the thought of using the Internet. | 34 | 41 | 23 | 13 | 26 | 23 | 7 | 11 | 0 | 0 |
| I feel at ease using the Internet. | 0 | 1 | 10 | 11 | 11 | 11 | 29 | 26 | 40 | 39 |
| The Internet is responsible for many of the good things we enjoy. | 1 | 2 | 10 | 9 | 20 | 16 | 33 | 30 | 26 | 31 |

## Appendix Table 4. Group Faciliator Debrief Questionnaire

Please share your thoughts and experiences about the **facilitation** of this weekly virtual group. The following questions will help to guide the discussion.

1. What are your views on the group guidelines for the weekly virtual group? Are they appropriate and sufficient? Are there any guidelines you would add or subtract?
2. Were there any technical issues that impacted your ability to run the group effectively?
3. Were there any challenges with managing the number of attendees during any of the weekly virtual groups?
4. The focus of the weekly virtual sessions was on the reflective exercise from that week’s module. What are your thoughts about how that format worked? What challenges did you face in terms of keeping the group focused on the reflective exercise task?
5. Are there any reflective exercises that you think could cause users to feel uncomfortable?
6. What is your view on possible risks for users of the format of the Trauma PORTAL intervention with the virtual modules and weekly groups? What about any possible risks for providers?
7. What are your thoughts on whether the Trauma PORTAL intervention will improve the quality of life of users?
8. Would you consider facilitating another weekly virtual group for the Trauma PORTAL intervention in the future? Why or why not?
9. Please share any other thoughts about any aspect of the group.

Appendix Table 5. Health Service Utilization Questionnaire (HSUQ) characteristics **of randomized study participants (N=181).**
Characteristics are shown as n (%) unless otherwise specified. *Not all participants completed this questionnaire.

| Statement | Timepoint | PORTAL (n=91) | Control (n=90) |
| --- | --- | --- | --- |
| Medical conditions? | Baseline | 64 (70%) | 67 (74%) |
| Taking meds, vitamins, or supplements? | Baseline | 81 (89%) | 81 (90%) |
| Changes in meds, vitamins or supplements? | Week 8 | 19 (21%) | 15 (17%) |
|  | Week 16 | 23 (25%) | 18 (20%) |
| Drink alcohol? | Baseline | 38 (42%) | 34 (38%) |
|  | Week 8 | 27 (30%) | 25 (28%) |
|  | Week 16 | 24 (26%) | 21 (23%) |
| Use cannabis? | Baseline | 25 (28%) | 25 (28%) |
|  | Week 8 | 16 (18%) | 20 (22%) |
|  | Week 16 | 19 (21%) | 15 (17%) |
| Receiving mental health treatments elsewhere? | Baseline | 48 (53%) | 48 (53%) |
|  | Week 8 | 34 (37%) | 36(40%) |
|  | Week 16 | 40 (44%) | 33 (37%) |
| Currently smoke cigarettes? | Baseline | 9 (10%) | 7 (8%) |
| Hospitalization in the past 8 weeks due to mental health reasons? | Baseline | N/A N/A |  |
|  | Week 8 | <5 (<5%) <5 (<5%) |  |
|  | Week 16 | <5 (<5%) <5 (<5%) |  |

# **Appendix Table 6. ACE score breakdown** of those who completed the measure (n =174). Scores are shown as n (%) unless otherwise specified.

| **ACE Score** | **PORTAL (n=88)** | **Control (n=86)** |
| --- | --- | --- |
| Score of 0 | 0 | 0 |
| Score of 1 | 4 (4.6) | 3 (3.5) |
| Score of 2 | 1 (1.1) | 4 (4.7) |
| Score of 3 | 9 (10.2) | 6 (7.0) |
| Score of 4 | 16 (18.2) | 12 (14) |
| Score of 5 | 9 (10.2) | 19 (22.1) |
| Score of 6 | 19 (21.6) | 15 (17.4) |
| Score of 7 | 17 (19.3) | 12 (14) |
| Score of 8 | 5 (5.7) | 5 (5.8) |
| Score of 9 | 7 (8.0) | 6 (7.0) |
| Score of 10 | 1 (1.1) | 4 (4.7) |

ACE scores range from 0–10, based on the original CDC–Kaiser Permanente Adverse Childhood Experiences questionnaire.

# **Appendix Table 7. Online self-paced module completion** among Trauma PORTAL participants (n=91).

| Number of modules completed | Participants (n) | % of total sample |
| --- | --- | --- |
| 0 | 3 | 3 |
| 1 | 5 | 6 |
| 2-3 | 6 | 7 |
| 4-5 | 5 | 6 |
| ≥ 6 | 72 | 79 |

# **Appendix Table 8. Attendance of Trauma PORTAL participants** at optional weekly virtual therapist-led group sessions (n = 91).

| Number of sessions attended | Participants (n) | % of total sample |
| --- | --- | --- |
| 0 sessions | 23 | 25 |
| 1 session | 14 | 15 |
| 2-3 sessions | 13 | 14 |
| 4-5 sessions | 13 | 14 |
| 6-8 sessions | 28 | 31 |

# **Appendix Table 9. Post-intervention clinical disposition of the Trauma PORTAL participants within the Trauma Therapy Program** (n = 91).

| **Summary of Participants’ post-study outcomes** | **PORTAL participants n (%)** |
| --- | --- |
| Met module completion threshold (≥75%) and proceeded to a further trauma therapy group in TTP | 69 (76%) |
| Met module completion threshold (≥75%) and elected to repeat Trauma PORTAL in a synchronous group format | 6 (7%) |
| Did not meet the completion threshold and were recommended to repeat Trauma PORTAL in a synchronous group format | 6 (7%) |
| Assessed as not appropriate for further trauma therapy in TTP at this time | 0 |
| Did not complete exit interview (missed appointment or voluntary withdrawal from TTP) | 10 (11%) |

Post-intervention disposition was determined through routine clinician-led exit interviews conducted as part of standard Trauma Therapy Program procedures and was not a prespecified study outcome.

## **Appendix Table 10.** Comparisons between Trauma PORTAL and control on the primary and secondary outcome measures (complete-case ANCOVA analysis)

| **Outcome Measure** | **Timepoint** | **PORTAL adjusted mean (95% CI)** | **Control adjusted mean (95% CI)** | **Adjusted mean difference (95% CI)** | **p value** |
| --- | --- | --- | --- | --- | --- |
| PTSD severity (PCL-5) | Week 8 | 39.5 (36.0 to 43.0) | 46.4 (42.8 to 50.0) | -7.0 ( -12 to –1.9) | 0.0005 |
|  | Week 16 | 35.6 (31.5 to  39.7) | 43.7 (39.5 to 48.0) | -8.1 ( -14 to -2.2) | 0.0043 |
| PTSD severity (CAPS-5) | Week 8 | 26.3 (23.0 to 29.6) | 29.7 (26.5 to 32.8) | -3.4 (-8 to 1.2) | 0.1763 |
|  | Week 16 | 24 (20.4 to 27.6) | 28.6 (25.2 to 32.0) | -4.6 (-9.6 to 0.4) | 0.0965 |
| Emotion Regulation (DERS) | Week 8 | 49.7 (46.6 to 52.8) | 53.9 (50.7 to 57.1) | -4.2 (-8.6 to 0.3) |  |
|  | Week 16 | 47.4 ( 44.2 to 50.6) | 54.0 (50.7 to 57.3) | -6.6(-11.2 to -2.0) | 0.0161 |
| Self-Compassion (SCS) | Week 8 | 2.6 (2.4 to 2.8) | 2.4 (2.2 to 2.6) | 0.2 (-0.03 to 0.5) |  |
|  | Week 16 | 2.8 (2.6 to 3.0) | 2.5 (2.3 to 2.7) | 0.3 (0.03 to 0.6) | 0.0572 |
| Depression (DASS-21) | Week 8 | 19.0 (16.2 to 21.7) | 22.9 (20.1 to 25.7) | -4.0 (-7.9 to -0.03) |  |
|  | Week 16 | 17.8 (14.9 to 20.8) | 20.9 (17.8 to 24.0) | -3.1 (-7.3 to 1.2) | 0.1537 |
| Anxiety (DASS-21) | Week 8 | 15.8 (13.6 to 18.0) | 19.3 (17.1 to 21.5) | -3.5 (-6.6 to  -0.4) |  |
|  | Week 16 | 14 (11.6 to 16.4) | 18.3 (15.8 to 20.8) | -4.3 (-7.8 to -0.8) | 0.1276 |
| Stress (DASS-21) | Week 8 | 20.3 (18.0 to 22.5) | 22.5 (20.2 to 24.8) | -2.2 (-5.4 to 1.0) |  |
|  | Week 16 | 19.8 (17.4 to 22.1) | 22.3 (19.9to 24.8) | -2.6 (-6.0 to 0.8) | 0.5074 |

## **Appendix Table 11.** Comparisons between Trauma PORTAL and control on the primary and secondary outcome measures (intention-to-treat, TimeXGroup ANCOVA analysis)

| **Outcome Measure** | **Timepoint** | **PORTAL adjusted mean (95% CI)** | **Control adjusted mean (95% CI)** | **Adjusted mean difference (95% CI)** | **p value** |
| --- | --- | --- | --- | --- | --- |
| PTSD severity (PCL-5) | Week 8 | 38.6 (35.7 to 41.4) | 45.4 (42.6 to 48.2) | -6.8 (-10.8 to -2.8) | 0.0007 |
|  | Week 16 | 36.0 (32.7 to 39.1) | 43.3 (40.0 to 46.5) | -7.3 (-11.8 to 2.8) | 0.0011 |
| PTSD severity (CAPS-5) | Week 8 | 27.2 (25.0 to 29.3) | 29.9 (27.8 to 32.1) | -2.7 (-5.8 to 0.3) | 0.026 |
|  | Week 16 | 24.3 (22.1 to 26.5) | 29.2 (27.0 to 31.3) | -4.9 (-7.0 to -1.8) | 0.0037 |
| Emotion Regulation (DERS) | Week 8 | 49.0 (46.5 to 51.5) | 53.2 (50.7 to 55.7) | -4.2 (-7.8 to -0.6) |  |
|  | Week 16 | 47.3 (44.8 to 49.8) | 53.1 (50.6 to 55.6) | -5.71 (-9.2 to -2.2) | 0.0269 |
| Self-Compassion (SCS) | Week 8 | 2.6 (2.5 to 2.8) | 2.4 (2.3 to 2.6) | 0.2 (0.006 to 0.4) |  |
|  | Week 16 | 2.8(2.6 to 2.9) | 2.5 (2.3to 2.6) | 0.3 (0.1 to 0.5) | 0.1525 |
| Depression (DASS-21) | Week 8 | 18.7 (16.5 to 20.9) | 21.9 (19.7 to 24.2) | -3.3 (-6.4 to – 0.1) |  |
|  | Week 16 | 17.4(15.1 to 19.7) | 20.5 (18.2 to 22.8) | -3.2 (-6.4 to 0.1) | 0.022 |
| Anxiety (DASS-21) | Week 8 | 15.4(13.6to 17.1) | 18.0 (16.2 to 19.8) | -2.7 (-5.2 to -0.1) |  |
|  | Week 16 | 14.2 (12.2 to 16.1) | 17.4 (15.5 to 19.4) | -3.3 (-6.0 to -0.6) | 0.1475 |
| Stress (DASS-21) | Week 8 | 19.9 (18.1to 21.7) | 22.4 (20.6 to 24.3) | -2.5 (-5.1 to 0.02) |  |
|  | Week 16 | 19.5(17.6 to 21.4) | 22.0 (20.1 to 23.9) | -2.5 (-5.1 to 0.2) | 0.3928 |

Appendix Table 12. Sensitivity analyses for complete-case ANCOVA.
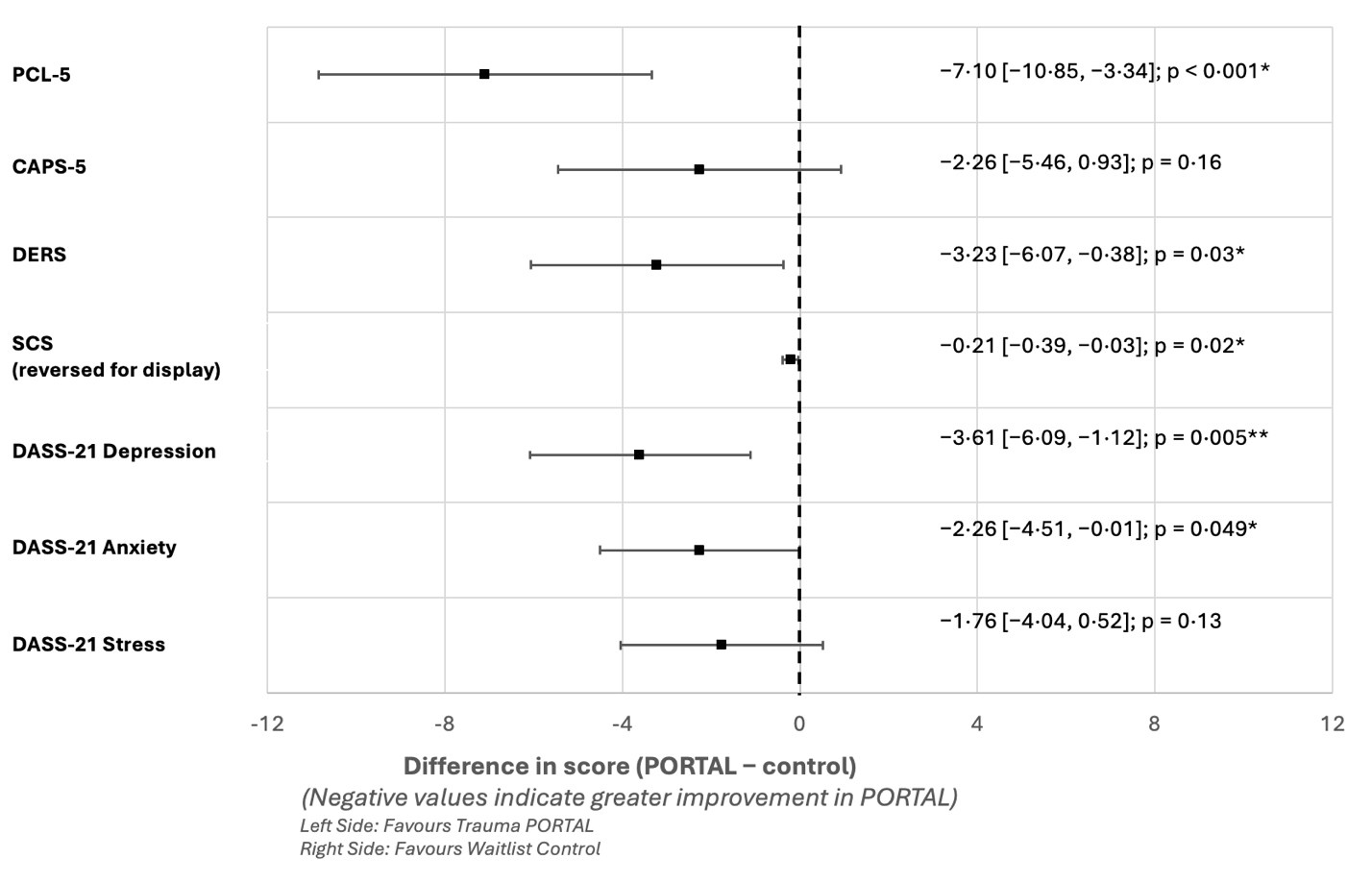


Figure 3. Points represent adjusted mean differences between PORTAL and control (PORTAL – control) with 95% confidence intervals. Negative values indicate greater improvement in the PORTAL group. For interpretive consistency, SCS scores were reversed so that higher self-compassion also appears as improvement to the left of zero. Asterisks denote significance levels (p < 0·05 =, p < 0·01 = *, p < 0·001 = ***).

Appendix Table 13. Participant responses to the Trauma PORTAL acceptability questionnaire **(n = 76).**

Note: Data represent the number of participants selecting each response option. Missing responses are indicated where applicable.

| **Statement** | **Strongly agree** | **Moderately agree** | **Mildly agree** | **Mildly disagree** | **Moderately disagree** | **Strongly disagree** |
| --- | --- | --- | --- | --- | --- | --- |
| The Trauma PORTAL has saved me time in that I did not have to visit Women's College Hospital or other healthcare professionals as often. | 45 | 16 | 11 | 1 | 2 | 1 |
| The Trauma PORTAL has increased my access to healthcare. | 35 | 20 | 16 | 3 | 1 | 1 |
| The convenience of being able to access the Trauma PORTAL on my own time was important to me. | 59 | 11 | 4 | 1 | 0 | 1 |
| The Trauma PORTAL was more comfortable than face-to-face therapy. | 25 | 17 | 20 | 5 | 6 | 3 |
| The Trauma PORTAL makes me worried about my privacy. | 0 | 6 | 8 | 7 | 15 | 40 |
| The Trauma PORTAL provided information that was helpful to me. | 50 | 20 | 5 | 0 | 0 | 1 |
| My problems are too complex or severe to benefit from the Trauma PORTAL. | 1 | 9 | 12 | 17 | 15 | 22 |
| I would recommend the Trauma PORTAL to other individuals with a history of childhood interpersonal trauma. | 55 | 11 | 8 | 1 | 0 | 1 |
| The Trauma PORTAL materials were difficult to understand. *1 missing response | 0 | 1 | 5 | 4 | 21 | 44 |
| After the Trauma PORTAL, I feel more prepared for further trauma therapy. | 38 | 27 | 8 | 1 | 2 | 0 |
| The Trauma PORTAL can/should be recommended to people in a similar condition to mine. | 51 | 13 | 9 | 0 | 2 | 1 |
| Participating in the Trauma PORTAL was a positive and supportive experience. | 43 | 20 | 8 | 2 | 2 | 1 |
| The optional video group meetings added value to the Trauma PORTAL. *2 missing responses | 40 | 9 | 15 | 5 | 4 | 1 |
| I was satisfied with the level of opportunity to participate in the video group meetings. *3 missing responses | 34 | 13 | 12 | 9 | 1 | 4 |
| I benefited from the involvement of the facilitators in the video group meetings. *4 missing responses | 44 | 9 | 7 | 7 | 3 | 2 |
| I benefited from the input of the group members in the video group meetings. *4 missing responses | 34 | 13 | 11 | 7 | 5 | 2 |
| I am concerned that the person who monitors my condition through the Trauma PORTAL does not know my personal health history. | 1 | 6 | 18 | 10 | 21 | 20 |
| The Trauma PORTAL platform is easy to use. | 55 | 18 | 2 | 0 | 0 | 1 |
| It takes a lot of effort to learn how to use the Trauma PORTAL platform. | 0 | 1 | 4 | 12 | 13 | 46 |
| The online Trauma PORTAL platform is an effective way to learn this material. | 35 | 27 | 8 | 3 | 1 | 2 |

# **Appendix Table 14. Responses to the optional Iterative Feedback Form (IFF)** completed by Trauma PORTAL participants. Each participant received one IFF per week over the 8 weeks of the study, with 55 unique responses submitted (7.6% of 648 possible weekly submissions). Open-text responses from the optional Iterative Feedback Form (IFF) were reviewed descriptively to identify common experiences and suggestions. Because responses were anonymous, variable in length, and collected for ongoing program monitoring rather than formal qualitative research, no thematic coding was conducted.

| **IFF question** | **Summary** |
| --- | --- |
| Weekly login | Of the 55 participants with available login data, 50 reported at least one login across the 8-week intervention period. |
| Weekly module completion | Most respondents completed the weekly modules and average completion rates ranged from 70% to 85%. |
| Time per week spent on module | Among respondents (n=175), 5.57% (n=10)% selected 0 minutes, 6.15% (n=11) less than 30 minutes, 41.34% (n=74) 30–60 minutes, 33% (n=59)1–2 hours, and 11.73% (n=21) % more than 2 hours. |
| What worked particularly well in the Trauma PORTAL intervention (modules or group session)? | In open-text responses to the Iterative Feedback Form, participants described the Trauma PORTAL intervention as engaging, flexible, and validating. The online modules were viewed as clear, well-paced, and accessible, with participants highlighting the usefulness of psychoeducation, self-reflection exercises,grounding strategies and coping tools. The weekly virtual groups were consistently identified as supportive, normalizing, and beneficial for integrating module content and reducing isolation. Many participants appreciated the combination of self-paced learning and facilitated discussion, describing the intervention as “empowering” and “helpful for understanding myself better. |
| What could have been improved this week in the Trauma PORTAL intervention (modules or group)? | The most frequent comments related to group structure and pacing, including requests for more clarity and direction around expectations for group participants, more time for discussion, and gentler support for quieter participants. A small number mentioned technical or logistical issues such as audio quality, access timing, or difficulty printing materials. |
| Barriers to completion on a weekly basis | On average, 2–3 respondents per week reported not having enough time to complete the modules. No respondents reported disliking the content or experiencing technical difficulties accessing the platform. One to two per week selected “Other,” without elaboration. |

Appendix Table 15. Facilitator Debrief Findings
To inform implementation and refinement of the Trauma PORTAL, semi-structured debriefs were conducted with group facilitators following each 8-week cohort. Three facilitators participated in a total of six debriefs. Given the small number of facilitator participants and the evaluative nature of the interview guide, we conducted a **descriptive qualitative content analysis** rather than an interpretive thematic analysis. Transcripts were reviewed to identify recurring observations across cohorts, structured according to the domains in the debrief guide. The aim was to summarise consistent patterns in facilitator experiences rather than develop conceptual themes.

| **Domain** | **Summary of Facilitator Observations** | **Illustrative Quotes** |
| --- | --- | --- |
| Group Guidelines and Participant Preparation | Guidelines were clear and appropriate. Late-joining participants sometimes required additional orientation. Weekly reminders supported clarity, and facilitators suggested brief early communication about the programme’s purpose and structure. | “The guidelines made sense, but late joiners needed a bit more support.”  “A brief reminder each week helped keep everyone aligned.” |
| Technical and Logistical Considerations | Technical challenges were minimal. Most issues involved delayed module access due to late registration, occasional confusion with Zoom links, or difficulty locating handouts. These were usually resolved by week two. | “There were no major tech issues; it was mainly delays in module access at the start.”  “After the first week, everything ran smoothly.” |
| Attendance and Group Size | Although approximately 25 participants enrolled per cohort, weekly attendance typically ranged from around 8 to 12 and was stable. Facilitators reported that this size supported balanced discussion and engagement. | “Around ten people felt like the right balance for conversation.”  “Once the group settled in, the attendance was consistent.” |
| Balance of Content and Reflection | Sessions focused primarily on participant reflections and questions. Structured reflective exercises were used less frequently, but reflection emerged naturally and provided depth. | “The group discussion itself became the main reflective anchor.”  “Participants came ready to talk about what they learned.” |
| Participant Benefits Observed | Facilitators noted strong engagement, meaningful application of skills, greater understanding of trauma-related patterns, and appreciation of the flexibility of self-paced modules. Participants used the group to integrate learning collaboratively. | “Participants often described the content as helping them make sense of their reactions.”  “They appreciated being able to pause and return to the videos.” |
| Emotional Activation and Pacing | Some participants experienced emotional activation while working through the modules. Facilitators emphasised the importance of guidance on pacing, pausing, and returning to material when ready. | “Certain topics were activating, but the pacing options helped people manage it.”  “Having the choice to pause was really important.” |
| Facilitator Experience | Facilitators described the sessions as meaningful, collegial, and less intensive than other trauma groups where the sessions are longer in length. Co-facilitation was important for managing flow and maintaining a supportive environment. | “Co-facilitation made a big difference in holding the space.”  “These groups were emotionally rich.” |
| Suggestions for Programme Refinement | Recommendations included clearer onboarding about technical aspects, weekly guideline reminders, extending module access, ensuring all materials were easy to locate, and continuing co-facilitation. | “A simple one-page overview of the process would reduce confusion.”  “Extending access to the modules would help people consolidate what they learned.” |

| **Outcome** | **Number of participants missing data at baseline,  n (%)** | **Number of participants missing data at week 8, n (%)** | **Number of participants missing data at week 16, n (%)** | **Number of complete cases for week 8 sensitivity analysis*,  n (%)** |
| --- | --- | --- | --- | --- |
| PCL-5 | 3 (2%) | 27 (15%) | 35 (19%) | 153 (85%) |
| CAPS-5 | 0 | 45 (25%) | 64 (35%) | 136 (75%) |
| DASS-21 | 3 (2%) | 27 (15%) | 35 (19%) | 153 (85%) |
| DERS-18 | 3 (2%) | 27 (15%) | 35 (19%) | 153 (85%) |
| SCS | 3 (2%) | 27 (15%) | 36 (20%) | 153 (85%) |

Appendix Table 16. Missing Data by Outcome
Note: Full sample is N=181

*Defined as the number of people with complete data at both baseline and week 8.
