# Supplementary material for: Efficacy of a hybrid psychoeducational and skills-based therapy (Trauma PORTAL) for adults with PTSD related to childhood interpersonal trauma: a parallel-group, randomised controlled trial
Source: eClinicalMedicine. 2026 Jun 5;96:104003. doi: 10.1016/j.eclinm.2026.104003 (PMC13266226; doi:10.1016/j.eclinm.2026.104003)
Supplement: Protocol [file mmc2.docx]

The Trauma PORTAL Project: A Randomized Controlled Trial

## **Research Protocol**

**Title:** The Trauma PORTAL Project: **A** **Randomized Controlled Trial of a Virtual Asynchronous Psychoeducational Psychotherapy Treatment for Survivors of Childhood Interpersonal Trauma**

**Funding:** Internal WCH Department of Psychiatry

**Principal Investigators:**

**Dr. Dana Ross**, MD, MSc, FRCPC

Psychiatrist, Trauma Therapy Program, Women’s College Hospital

Assistant Professor, Department of Psychiatry, University of Toronto

Email: [dana.ross@wchospital.ca](mailto:dana.ross@wchospital.ca); Telephone: 416-323-6400 ext. 6585

**Dr. Nancy McCallum**, MD, MSc, FRCPC

Psychiatrist, Program Lead, Trauma Therapy Program, Women’s College Hospital

Assistant Professor, Department of Psychiatry, University of Toronto

Email: [nancy.mccallum@wchospital.ca](mailto:nancy.mccallum@wchospital.ca); Telephone: 416-323-6400 ext. 4937

**Co-Investigators:**

**Dr. Simone Vigod**, MD, MSc, FRCPC

Chief, Department of Psychiatry, Women’s College Hospital

Professor, Department of Psychiatry, University of Toronto

Email: [simone.vigod@wchospital.ca](mailto:simone.vigod@wchospital.ca); Telephone: 416-323-6400 ext. 4080

**Tina Behdinan**, MD, MSc

PGY-4 Resident in Psychiatry, University of Toronto

Department of Psychiatry, Faculty of Medicine

**Sophie Soklaridis**, PhD
Scientist – The Wilson Centre
Independent Scientist and Section Head of Education
Research- Centre for Addiction and Mental Health
Associate Professor- Departments of Psychiatry and Family & Community Medicine,
Temerty Faculty of Medicine, University of Toronto

**David Rojas**, PhD

Scientist, The Wilson Centre

Director, Program Evaluation, Office of Assessment & Evaluation

MD Program, Temerty Faculty of Medicine

Assistant Professor, Department of Obstetrics & Gynaecology, University of Toronto

**Mahum Musheer**, MED, RP

Registered Psychotherapist

Trauma Therapy Program, Women’s College Hospital

**Abstract:**

Virtual delivery of mental health care is an emerging strategy for increasing access and enhancing delivery of mental health services; however, evidence that virtual interventions are an efficacious form of therapy is limited. An asynchronous virtual treatment program that allows patients to access program material at any time could be a widely accessible, cost-effective alternative to in-person or synchronous virtual group therapy. The Trauma Therapy Program (TTP) at Women’s College Hospital (WCH) follows clinical guidelines for the treatment of complex post-traumatic stress disorder that recommend a staged approach to treatment for adults suffering from the sequelae of childhood interpersonal trauma (CIT). The initial stage is safety and stabilization; in TTP, this begins with the Resourced and Resilient (R&R) group, a stage 1 trauma-focused psychoeducational psychotherapy group. Psychoeducational psychotherapy is a widely used approach to help patients understand the impact of their trauma history, challenge maladaptive behaviour patterns, learn safer coping skills and reduce trauma related symptoms as part of a comprehensive approach to the treatment of PTSD. In order to address gaps in equitable access to trauma-focused care, the investigators developed the Trauma PORTAL: Providing Online tRauma Therapy using an Asynchronous Learning platform. We developed an asynchronous virtual multimedia version of R&R consisting of 8 modules, called the Trauma PORTAL; previously called electronic Resourced and Resilient or e-R&R. We then conducted an open-label pilot study where we offered the the Trauma PORTAL intervention that includes access to the asynchronous virtual modules, along with an optional weekly 1-hour synchronous virtual group that offers patients an opportunity to ask questions about content from the modules. Preliminary data from our open-label eR&R pilot study demonstrates feasibility, usability, and acceptability, as well as a significant improvement in PTSD symptoms from pre- to post-group. This phase of the Trauma PORTAL project seeks to test the efficacy and further assess the intervention processes including recruitment, retention, acceptability, and adherence to the Trauma PORTAL intervention through a randomized controlled trial.

**Location:** Trauma Therapy Program, Women’s College Hospital

**Research Ethics Approval:** Approval is being sought from Women’s College Hospital.

**1.0 Background**

**1.1 Childhood Interpersonal Trauma.** Childhood interpersonal trauma is prevalent and underrecognized in our society^1^. In Canada, approximately 1 in 3 adults have experienced childhood trauma. Childhood interpersonal trauma is known to have adverse effects on victims well into adulthood, including the development of serious mental illness, substance abuse, suicidality, chronic physical conditions, disability, and revictimization later in life^2–6^. The 2003 report to Law Commission of Canada estimates the socioeconomic burden of child abuse in terms of healthcare, social services, education, judicial system, employment, and personal costs, to over 15 billion dollars annually^7^. Given the significant personal and societal impacts, many have advocated for greater resources to be dedicated to addressing childhood interpersonal trauma and its sequelae^2,3,8^.

**1.2 Management of Sequelae of Childhood Interpersonal Trauma.** Management of childhood interpersonal trauma sequelae involves a validated staged approach to trauma recovery^9^. Stage I trauma treatment focuses on safety and stabilization. Individuals impacted by childhood interpersonal trauma often have difficulty with emotion regulation due to prolonged and repeated experiences of feeling unsafe in interpersonal relationships and being raised in environments without modelling of safe and healthy methods of coping with distress. As a result, these individuals often find themselves in unstable/unsafe interpersonal relationships as adults and use unhealthy coping behaviours in times of distress. The aims of Stage I treatment are therefore to develop an understanding of patterns and behaviours in response to trauma, establish a safe environment in which to process psychoeducational material, and improve emotion regulation with development of interpersonal skills and healthy coping strategies. At Women’s College Hospital (WCH) in Toronto, Ontario, Stage I treatment is delivered via the Resourced & Resilient (R&R) group through the Trauma Therapy Program (TTP). R&R is a trauma-focused psychoeducational psychotherapy group that utilizes accepted principles in the approach to early stage trauma recovery. Stage II treatment is focused on remembrance and mourning, which asks the survivor of childhood interpersonal trauma to tell their trauma story and allow themselves to experience and process the grief that surfaces throughout this process. The goal of Stage II treatment is for the survivor to reclaim their trauma story and reconceptualize it as being only one part of their life’s narrative. Stage III treatment involves reconnecting with the self and others, along with rebuilding life in the present and pursuing future aspirations. Importantly, not all survivors of childhood interpersonal trauma require or choose to complete later-stage trauma treatment. Some survivors may experience significant improvement with Stage I treatment only, while others with more complex presentations may need to proceed to Stage II and/or Stage III treatment.

**1.3 Access to Care for Childhood Interpersonal Trauma.** There are very few resources for trauma treatment across all stages in Ontario, nationally, and globally. Lack of access leads to growing waitlists for services, increased burden on other areas of the healthcare system, and delayed recovery of childhood trauma survivors. Furthermore, delayed recovery leads to worsening of symptoms that may have been adequately addressed with Stage I treatment. As a result, symptoms increase in severity and chronicity, which ultimately necessitates higher stage trauma treatment that may have otherwise been avoided. Importantly, delivery of Stage I treatment does not necessarily require highly specialized therapist training as it involves a large psychoeducational and skill-based component. Therefore, there is a rationale for finding more accessible ways to deliver Stage I treatment than with 1:1 highly-specialized trauma therapist-patient settings. For the past 12 years, TTP at WCH has been delivering Stage I treatment via in-person group format (8 weeks), and has established a clear manualized set of materials and training for therapists delivering the intervention known as the Resourced and Resilient group (R &R). Each R&R groups can accommodate a maximum of 12 patients and require 2 TTP facilitators. During the pandemic, the program pivoted to a virtual synchronous group format for the delivery of R&R. At present, the TTP receives ~100 referrals per month for Stage I treatment (~1200 referrals per year) but is resourced to provide R&R to a maximum of 360 individuals per year (which is equivalent to 750 hours of therapist time). The demand for these services results in wait times of up to 1 year and growing. Furthermore, the growing waitlist reduces the program’s capacity to provide more specialized later-stage trauma therapy for individuals who have completed R&R.

**1.4 Electronic Health (eHealth) for Psychological Treatments.** Electronical heath (eHealth) interventions are being used with increasing frequency for a wide range of psychological treatments in mental health to improve access, efficiency, and ultimately effectiveness of care^10,11^. Treatments that can be standardized and are focused on skills training are particularly suited to dissemination via eHealth platforms^12^. Benefits of eHealth treatments in previous studies include reduced mental health care disparities by allowing individuals with geographic limitations, challenging work schedules, and disability to access treatment^13^. They may also an effective and viable treatment option for individuals with mild psychiatric symptoms, thereby reserving specialized services within the healthcare sector for individuals with severe or refractory psychiatric symptoms^10,14^. Specifically, eHealth interventions have been associated with a reduction in PTSD symptoms^11,15^. One example is PTSD Coach, a mobile health intervention with self-assessment tools, coping skills for PTSD symptoms, and psychoeducation^16^. Other examples include web-based CBT programming (PTSD Online, From Survivor to Thriver) as well as exposure protocols (Interapy, DE-STRESS)^11,15^. To address this growing demand, we propose an innovative system to deliver the R&R Stage I treatment in an asynchronous virtual format (i.e. a web-based intervention with psychoeducational course material that can be completed independently, and an optional 60-minute weekly online group session to review course material) to individuals on the waitlist for the TTP at WCH (The Trauma PORTAL intervention).

**1.5 Work to Date.** The TTP clinical team at WCH has been delivering the in-person R&R group for many years, and recently pivoted to synchronous virtual R&R groups during the pandemic. The R&R curriculum and materials are already manualized in print-form. The Trauma PORTAL research team at WCH has already completed Phase 1 of this project involving the initial design and development of the virtual modules by investigators, and then iterative changes with input from healthcare professionals and patient participants. Phase 2) An Open Label Pilot Study of eR&R assessed the feasibility, usability and acceptability of eR&R, allowing for adjustments to be made to the intervention based on patient and provider feedback. Sixty-seven TTP patients who were on the wait list for R&R were enrolled in the eR&R intervention. Preliminary data suggests that the eR&R intervention is feasible, usable and acceptable to patients, and our secondary outcome measures indicates a clinically significant improvement in PTSD symptoms. The proposed study, Phase 3, will consist of a randomized controlled trial comparing outcomes of individuals randomized to immediate treatment with the Trauma PORTAL intervention to those on a waitlist to be treated. The primary outcome will be symptoms of post-traumatic stress disorder immediately post-intervention. Secondary outcomes will be additional clinical measures, and process measures that will help to inform future spread and scale, including issues related to recruitment, retention, and intervention acceptability and adherence.

**2.0 Study Design**

**2.1 Objectives**

In this third phase of the Trauma PORTAL research project, we aim to conduct a randomized controlled trial to determine the efficacy of the Trauma PORTAL intervention. We specifically aim to measure clinical symptoms to generate estimates of the effect of the Trauma PORTAL intervention on clinical measures of PTSD, depression, anxiety, and emotion regulation compared to a care-as-usual condition. We will also evaluate the intervention processes, including recruitment, retention, acceptability and adherence to inform spread and scale post-trial if the intervention is demonstrated to be effective.

**2.2 Trial Design**

Individuals will be recruited from the waitlist for our “live” synchronous virtual or in-person R&R group in the Trauma Therapy Program (TTP). The Trauma Therapy Program is a specialized trauma-focused therapy service for persons with childhood histories of trauma. The program offers confidential, time-limited and primarily group-based psychotherapy to adults who have experienced childhood interpersonal trauma including physical, sexual, emotional abuse and/or neglect that occurred between the ages of 0-18. The TTP is composed of an interdisciplinary team of trauma therapists with backgrounds in psychiatry, psychotherapy, psychology, social work, and nursing.

Individuals will be randomized to receiving the Trauma PORTAL intervention (immediate treatment condition, ITC) or treatment as usual while on a waitlist (care-as-usual condition, CUC). Participants randomized to CUC will remain on the waitlist for live R&R **and** complete clinical measures at the same timepoints as the ITC group. We have elected NOT to use in-person or “live” online R&R as a comparator group. It is not expected that an online asynchronous therapist-facilitated platform would be non-inferior to a live group given that the relational component of a live group may be an important added therapeutic aspect. As such, it is difficult to hypothesize how well the Trauma PORTAL intervention would perform in relation to a live counterpart. Furthermore, there are many individuals for whom virtual asynchronous format of the Trauma PORTAL may be more accessible because of geographical distance from WCH or lack of flexibility with work scheduling, and therefore would not be appropriate for randomization to a live R&R group. Therefore the research team has decided that it is most ethical to conduct a trial with a care-as-usual control group.

**Figure 1: The Trauma PORTAL intervention RCT Data Collection Schema**


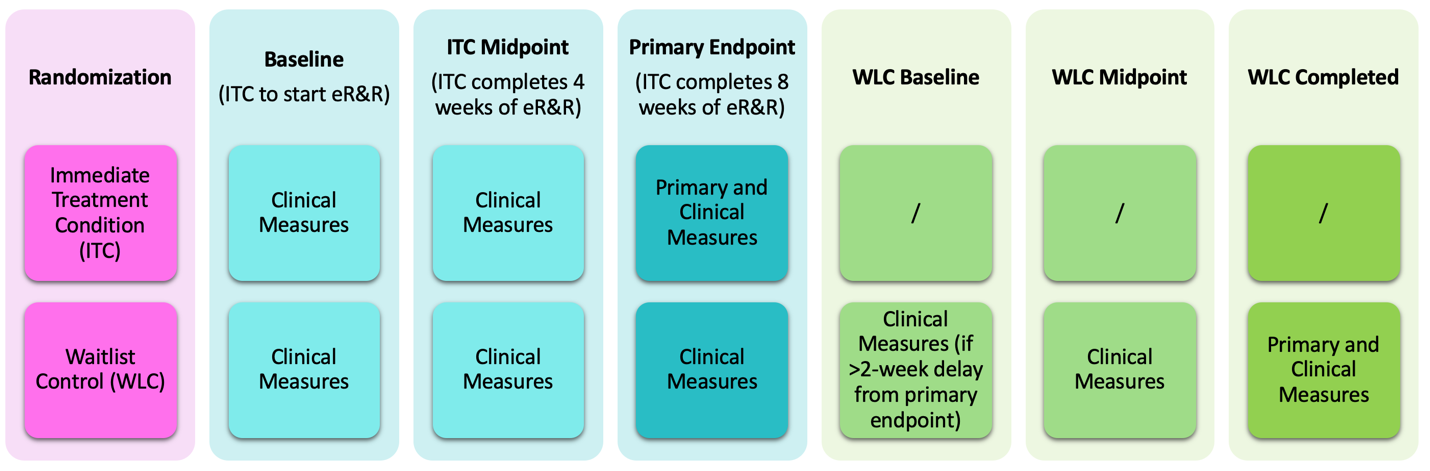

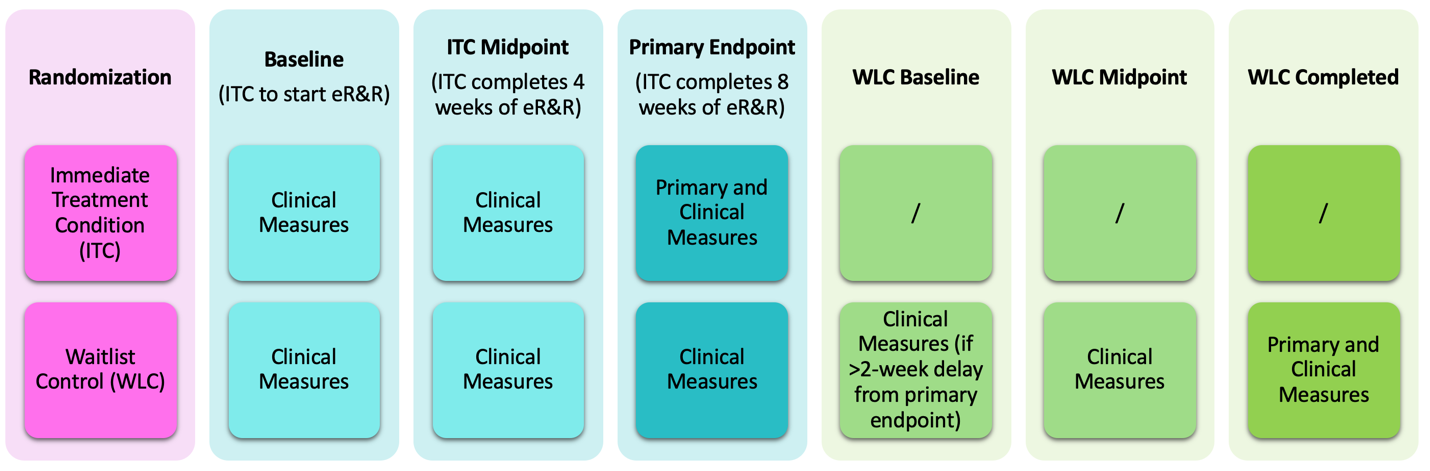

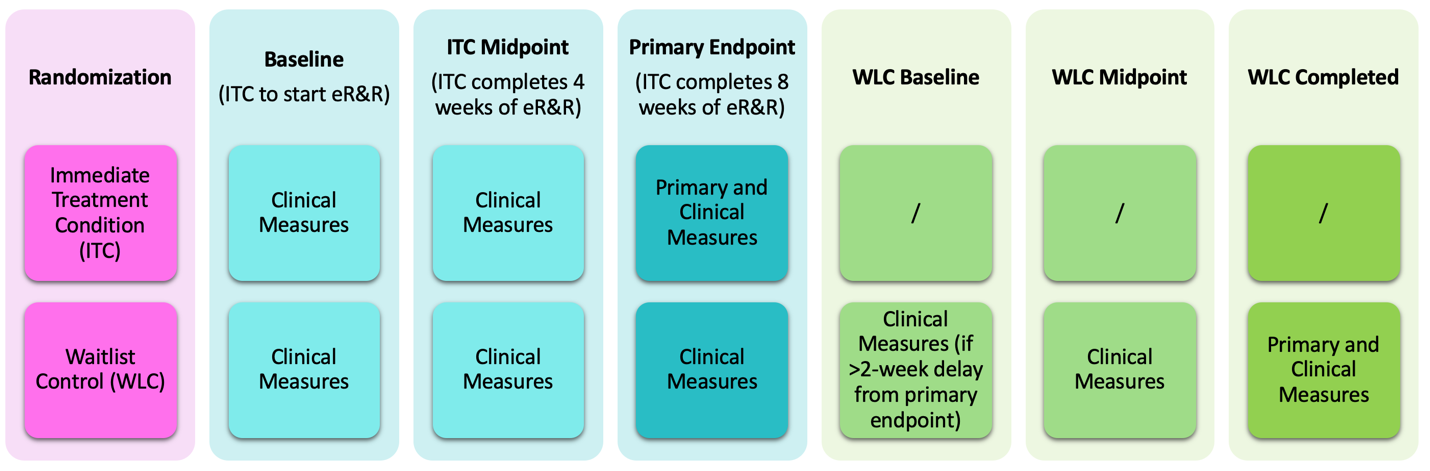


**Secondary Endpoint** (16-week mark)

Clinical

Measures

Care-as-Usual Condition (CUC)

Clinical

Measures

**2.2.1 Specific Research Questions**

***Primary Question***. What is the effect of an asynchronous trauma therapy intervention (Trauma PORTAL) compared to care-as-usual control, on posttraumatic stress disorder symptoms (measured using the self-report measure called the PTSD Checklist for DSM-5 (PCL-5) immediately post-intervention?

***Secondary Questions***. What is the effect of the Trauma PORTAL, compared to the care-as-usual control on participants’ PTSD symptoms as measured by the CAPS-5 scale, depressive and anxiety symptoms (measured using the Depression and Anxiety Stress Scale, DASS-21), emotion-regulation (measured using the Difficulties in Emotion Regulation Scale, DERS-18), and self-compassion (measured using the Short Self-Compassion Scale, Short SCS) immediately post-intervention?

***Health Service Questions*.** What processes of the Trauma PORTAL intervention relate to its efficacy and future scalability?

**2.3 Trial Interventions**

**2.3.1 Experimental Condition (the Trauma PORTAL intervention)**. The Trauma PORTAL is an innovative system to deliver the R&R Stage I trauma treatment in a virtual format to individuals with childhood interpersonal trauma.

The Trauma PORTAL R&R project is an 8-week virtual intervention. There are 2 main components:

1. **Online Course Platform:** All participants will be able to login to the Trauma PORTAL platform at any time of day (i.e. it will be open 24/7) to view materials, which include reading materials, video and audio clips, animated videos, podcasts, optional quizzes, audio-guided grounding skills and strategies, and self-reflective exercises. The content available to participants is similar to the in-person R&R groups. Each module consists of psychoeducational handouts, videos, interactive exercises, and discussion topics that has been adapted from the R&R curriculum (summarized in Table 1). Participants will have 9 weeks to complete the 8 modules which each take on average, 45 minutes to complete. They will also receive a pdf of the handouts from the modules which they can use to take notes or refer back to in the future.

We will communicate to participants early on that they must complete >75% of the modules to move onto a more advanced Stage I Trauma Therapy Group; otherwise, they must repeat a regular R&R group in order to stay in the TTP. If participants have not completed any of the modules (0% completion) by week 2 of the intervention, a research staff member will contact the participant to check in and ensure they know how to access the course.

1. **Virtual Weekly Group Session**: There will be an optional 60-minute weekly virtual group session for participants to engage with other participants in their group, and for group facilitator providers to discuss the materials covered, and answer any questions. Participants will log in to myHealthRecord and will join a Zoom video group visit to access the group. Each weekly group will be facilitated by 2 clinicians (group facilitator providers) specializing in trauma-focused therapy within the WCH Trauma Therapy Program (TTP) who have extensive experience with in-person and virtual synchronous R&R.


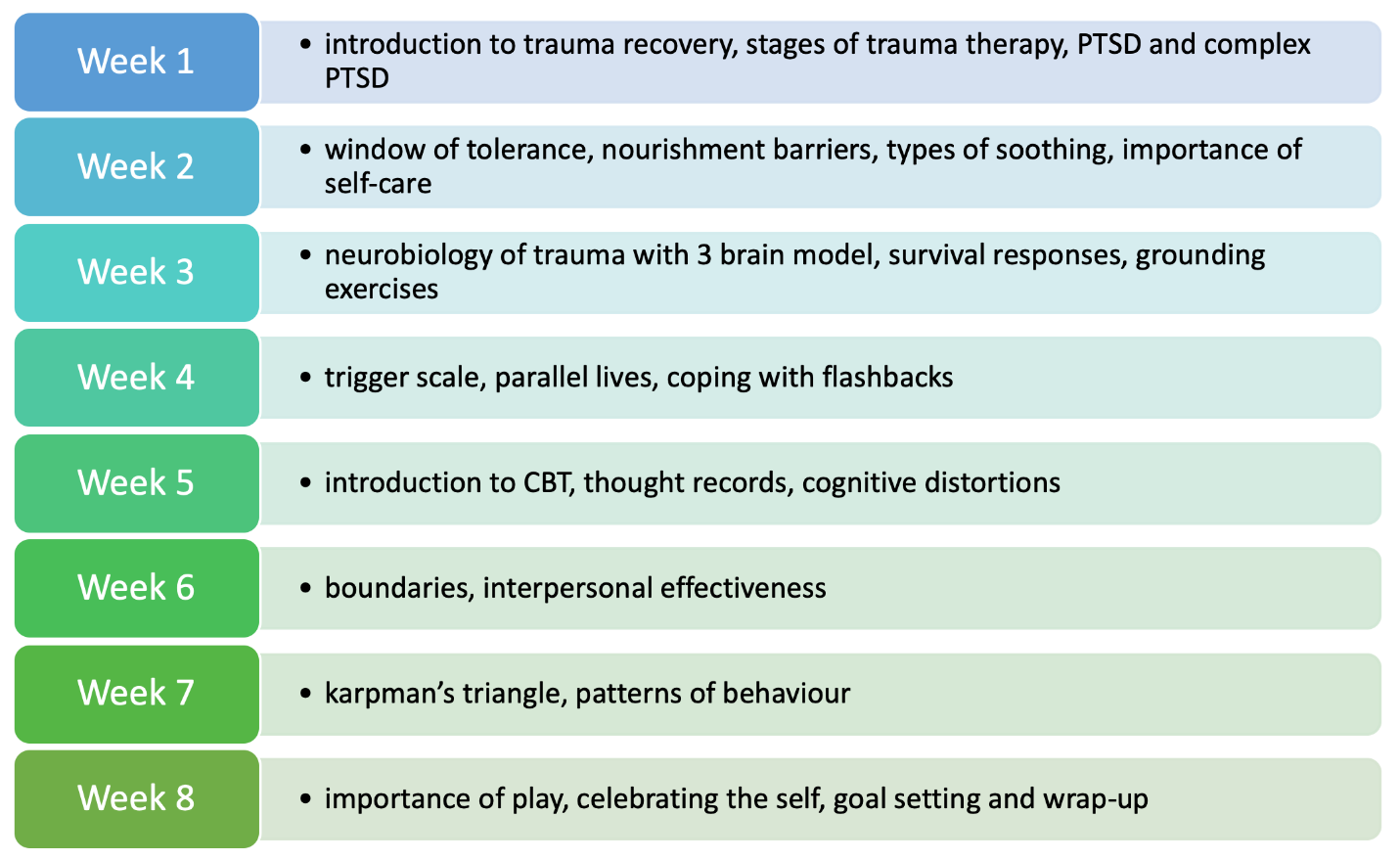


**Table 1: Overview of the Trauma PORTAL Weekly Module Content (subject to change - the order of the content based on iterative updates to the modules)**

**2.3.2 Control Condition**

A control group is needed for this study to isolate the effects of the Trauma PORTAL intervention on clinical symptoms from the effects of time. Individuals in the CUC group will be asked to complete clinical measures at timepoints corresponding to the ITC group’s baseline (prior to starting week one) and post-intervention (i.e. primary endpoint, end of week 8). Both groups will have two weeks to complete clinical measures at the timepoint that the ITC group is completing their post-intervention clinical measures, and then both groups will complete the clinical measures eight weeks later (16-week timepoint).

**2.4 Randomization and Blinding**

**2.4.1 Randomization**

After completing the baseline measures with the participant, the research assistant will randomize participants to the ITC or CUC group using REDCap^TM^ . After randomization is complete, the research assistant will assign a study ID to each participant, and if in the ITC group, inform them of their group start date and provide their login information to access the Trauma PORTAL platform via email. Participants will be given a clear timeline of how randomization affects them when they receive information about the Trauma PORTAL project during the informed consent discussion and once enrolled in the study via the Trauma PORTAL handbook, which includes pictorial instructions for logging onto the course platform.

**2.4.2 Blinding**

Team members analysing the data will be blinded to which group a participant’s study ID is linked in order to avoid bias when conducting between-group statistical analysis of clinical measures at the primary endpoint. While research assistants and patients will not be blinded to their randomization status, all clinical measures except the CAPS-5 will be collected via REDCap^TM^ in self-report questionnaires, which will remove investigator/clinician bias. Research staff members will conduct the CAPS-5 interview and will remain blinded to group assignment. Co-PIs may be unblinded to group assignment and randomize participants via REDCap. Co-PIs will not conduct clinical measures at timepoints after randomization if they are unblinded to groupings. TTP therapists will be Trauma PORTAL group providers and conduct the exit interviews.

**2.5 Eligibility Criteria**

This study is open to individuals referred to TTP who are accepted into the program, have attended the program orientation session, and are on the waitlist for our standard R&R group (in-person or synchronous virtual). Eligibility criteria for the study therefore overlap with criteria used to assess patient readiness for TTP, which is the reasoning behind having a TTP clinician conduct a clinical interview with the participant to confirm eligibility after the baseline research eligibility assessment.

**2.5.1 Inclusion Criteria**

- Aged > 18 years old
- Self-report childhood interpersonal trauma (physical, sexual, emotional, neglect) prior to age 18
- A diagnosis of PTSD aligned with the Mini International Neuropsychiatric Interview Module H
- Access to appropriate device and internet connection to access the intervention
- Attended an orientation session for the Trauma Therapy Program at WCH
- Suitable for Trauma PORTAL intervention (confirmed by clinical assessment with a TTP Therapist)*

**Participants will not be included if, based on a clinical assessment with a TTP therapist, there is a concern that they have: (1) significant difficulty with self-regulation which make them unsuitable for an outpatient asynchronous intervention, (2) cognitive impairments that would impede understanding and processing of educational material, (3) significant case management needs that would result in lack of suitability for asynchronous online group therapy, or (4) for any other clinical reason, at the discretion of the assessing TTP therapist.*

**2.5.2 Exclusion Criteria**

- Active alcohol or substance use disorder in past 3 months, as assessed by a score of 3 or 4 in items b-e on the GAIN-SS
- Active symptoms of mania or psychosis, or active suicidal ideation, as assessed by the MINI neuropsychiatric interview and the Columbia Suicidality Severity Scale
- Psychiatric hospitalization in the past 6 months
- Unable to read and understand English (intervention not yet translated)
- Previously completed an R&R group with an approved provider

**2.5.3 Stopping Criteria**

Participants may discontinue participation in the Trauma PORTAL intervention if there is a change in their condition that either meets exclusion criteria or they self-identify that that do not wish to continue in the study. Participants will be reminded that they can choose to stop participating at any point in the study, at which time the intervention would be discontinued. They can remain on the TTP waitlist for future services in TTP if appropriate based on TTP criteria and clinical assessment by the TTP clinical treatment team.

**2.6 Study Measures**

**2.6.1 Primary Outcome Measure**

We specifically aim to measure clinical symptoms to generate estimates of the effect of the Trauma PORTAL intervention on clinical measures of PTSD compared to a care-as-usual control condition. This will be measured using:

1. PTSD Checklist for DSM-5 (PCL-5). The PCL-5 is a 20-item self-report measure that assesses the 20 DSM-5 symptoms of PTSD. The wording of PCL-5 items reflects both changes to existing symptoms and the addition of new symptoms in DSM-5, updated from the original PCL. The self-report rating scale is 0-4 for each symptom, reflecting a change from 1-5 in the DSMIV version. Rating scale descriptors are the same: "Not at all," "A little bit," Moderately," "Quite a bit," and "Extremely." A total symptom severity score (range - 0-80) can be obtained by summing the scores for each of the 20 items. DSM-5 symptom cluster severity scores can be obtained by summing the scores for the items within a given cluster, i.e., cluster B (items 1-5), cluster C (items 6-7), cluster D (items 8-14), and cluster E (items 15-20)^19^. A provisional PTSD diagnosis can be made by treating each item rated as 2 = "Moderately" or higher as a symptom endorsed, then following the DSM- 5 diagnostic rule which requires at least: 1 B item (questions 1-5), 1 C item (questions 6-7), 2 D items (questions 8-14), 2 E items (questions 15-20). Evidence for the PCL for DSM-IV suggests that a 5-10 point change represents reliable change (i.e., change not due to chance) and a 10-20 point change represents clinically significant change.

**2.6.2 Secondary Outcome Measures**

We aim to measure clinical symptoms to generate estimates of the effect of the Trauma PORTAL intervention on clinical measures of clinician-assessed PTSD symptoms, and depression, anxiety, and emotion regulation compared to a care-as-usual control condition. We will also evaluate the intervention processes, including recruitment, retention, acceptability and adherence to inform spread and scale post-trial if the intervention is demonstrated to be effective. Clinical symptom scales include:

1. Clinician-Administered PTSD Scale for DSM-5 (CAPS-5). The CAPS-5 is a 30-item structured interview that can be used to make a current (past month) or lifetime diagnosis of PTSD and assess PTSD symptoms over the past week. “The assessor combines information about frequency and intensity of an item into a single severity rating […] [S]ymptom cluster severity scores are calculated by summing the individual item severity scores for symptoms corresponding to a given *DSM-5* cluster: Criterion B (items 1-5); Criterion C (items 6-7); Criterion D (items 8-14); and, Criterion E (items 15-20). A symptom cluster score may also be calculated for dissociation by summing items 19 and 20.
   - At least one Criterion B symptom
   - At least one Criterion C symptom
   - At least two Criterion D symptoms
   - At least two Criterion E symptoms
   - Criterion F is met (disturbance has lasted one month)
   - Criterion G is met (disturbance causes either clinically significant distress or functional impairment)”^31^
2. Depression and Anxiety Stress Scale (DASS-21). The DASS-21 is a self-reported 21-item questionnaire that assesses 3 subscales: depression (low positive affect, hopelessness, and anhedonia), anxiety (panic and physiological arousal) and stress (high negative affect). Respondents rate each item to reflect how much it applies to their experience over the preceding week on a Likert scale ranging from 0 (“did not apply to me at all”) to 3 (“applied to me very much”). The DASS is quick to complete and is responsive to treatment-related changes. It has good test-retest reliability, high internal consistency, and adequate convergent and discriminant validity with other measures of anxiety and depression^20^. It is a shortened version of a 42-item scale, so scores are multiplied by 2 in order to compare to reference ranges.
3. Difficulties in Emotion Regulation Scale (DERS-18). The DERS-18 is a multi-dimensional assessment of emotion regulation and dysregulation. It consists of a self-reported 18-item questionnaire that assesses 6 subscales: lack of awareness of one’s emotions, lack of clarity about the nature of one’s emotions, lack of acceptance of one’s emotions, lack of access to effective emotion regulation strategies, lack of ability to engage in goal-directed activities during negative emotions, and lack of ability to manage one’s impulses during negative emotions. Each subscale consists of 3 items, which are rated on a scale of 1 (almost never) to 5 (almost always). Three of the items are reverse-coded for scoring before totaling subscale and overall scores. While there are no official clinical cut-offs for the DERS-18, it is a well-validated and reliable scale that is used to assess changes in one’s ability to regulate emotion over time^21^.
4. In addition, participants will complete a validated questionnaire assessing self-compassion, the **Short Self-Compassion Scale**^30^ (Short SCS) by Dr. Kristen Neff. The Short Self-Compassion Scale is a 12-item survey assessing 6 subscales: self-judgement, self-kindness, common humanity, isolation, mindfulness, and overidentification. Questions are rated on a 5-point scale ranging from 1 (almost never) to 5 (almost always) with subscale scores computed by calculating the mean of subscale item responses. The total SCS score is computed by reverse scoring the negative subscale items (self-judgement, isolation, and overidentification) and then computing a total mean. Higher scores indicate higher levels of self-compassion. The short SCS has near perfect correlation to the long SCS and is a well-validated and reliable psychometric tool to measure the relationship between self-compassion and positive psychological health.

All clinical measures will be assessed at baseline,immediately post-intervention (i.e. after completion of the Trauma PORTAL intervention), and 8 weeks after the ITC group completes the intervention (i.e. secondary endpoint). Prior to their start date, individuals in the CUC group will also be asked to complete these measures at timepoints corresponding to the ITC group’s baseline,post-intervention (i.e. primary endpoint), and 16-week timepoint (i.e. secondary endpoint). All clinical measures except the CAPS-5 are self-reported. To complete the self-report measures, participants will be sent a link to secure surveys through REDCap^TM^. The CAPS-5 will be assessed at the three timepoints (at baseline, post-intervention, and at the 16-week timepoint).

**2.6.3 Other Measures**

To contextualize findings generated by the study, we will collect baseline information on participant demographics, and mental health service use, using the **Health Service Utilization Questionnaire**. The Health Service Utilization Questionnaire will be collected pre- and post-intervention and at the 16-week timepoint to capture any changes that could affect outcomes over the course of the Trauma PORTAL intervention. Participants will also complete a validated baseline questionnaire measuring their beliefs and attitudes about using the Internet via the **General Internet Attitude Scale**^24^. This scale will provide valuable information regarding study participants’ levels of comfort with the Internet, which will likely affect acceptance of an eHealth intervention such as the Trauma Portal intervention by participants.

- - 1. **Health Service Research Question Measures – Data for Scale and Spread**

1. *Recruitment*: Number of individuals interested in the study, proportion of interested individuals meeting eligibility criteria, proportion of eligible individuals who consent to study participation, reasons for non-consent to study participation, number of days required to fill each “participant group” (i.e. up to 25 participants)
2. *Adherence*: Rates of weekly login and module completion, as well as overall Trauma PORTAL completion, Number of weekly live synchronous groups attended, Length of time spent on weekly modules, Reasons for non-use or discontinuation of the intervention, Completion of baseline and post-intervention measures
3. *Safety:* Number of safety concerns, including need for trial psychiatrist to assess and/or assume care of the participant, and adverse events (i.e. participants meeting stopping criteria)
4. *Health system implications*: At the end of the intervention, we will conduct a **clinical chart review** using EPIC. Information collected include identifying the number of weekly online group sessions attended, if any additional contacts were required throughout the course of the intervention, and results of the assessment for next steps (classified as follows: (1) No further trauma therapy required; (2) Suitable for proceeding to more advanced Stage I Trauma Therapy Group; (3) In-person or “live” online R&R recommended for further foundational Stage I Trauma Therapy; or (4) Not suitable for further trauma therapy at this time.
5. *Acceptability of intervention in the target population*

Acceptability will be measured as follows:

- After completion of the Trauma PORTAL intervention, participants will be asked to complete an **Intervention Acceptability Questionnaire** comprised of open and closed ended questions. Closed ended questions will elicit feedback on (1) usability of the Trauma Portal; (2) benefits of the Trauma PORTAL intervention including usefulness and increased accessibility of healthcare; (3) perceived security of personal health information; (4) concerns regarding continuity of care; and (5) overall user satisfaction^17,18^.
- Participants will also be encouraged to report any issues with the website, as well as anything that needs to be changed in real time to the weekly group facilitator providers or the research team. To operationalize this, every week a short but optional **Iterative Feedback Form (IFF)** will be sent to participants via REDCap^TM^. The form will have two sections; the first prompting participant feedback: 1. “What worked particularly well this week in the Trauma PORTAL intervention?” and 2. “What could have been improved this week in the Trauma PORTAL intervention?”. Second, there will be short adherence metrics questions for participants to let us know how long they spent on each module, and reasons for not completing it (if applicable). A statement will be included that reminds participants to contact the RA if they no longer wish to continue in the study. These forms will be reviewed by the research team on a weekly basis.
  - 1. **Measurement Timing and Administration**

Participant flow is shown below, along with the timing of administration of the measures. All assessments completed with research staff will be completed via phonecall. All self-report measures will be completed online through a link provided by the research staff via email.

**Figure 2: RCT Participant Flow**

**Figure 3: Timeline of Outcome Measures**

|  | Baseline | Weekly | Post-Treatment | Long-Term | Use |
| --- | --- | --- | --- | --- | --- |
| Eligibility | Eligibility Q includes MINI modules C and L to rule out suicidality and psychosis, MINI module H to provide diagnosis of PTSD, and GAIN-SS to rule out active addiction | | |  | R |
| HSU | x |  | x | x | Co |
| GIAS | x |  |  |  | Co |
| ACE | x |  |  |  | Co |
| Chart Review** | Chart review data retrieved through EPIC after study completion. Includes # of sessions attended, exit interview results and any additional contact with other services throughout intervention | | |  | E |
| PCL-5 | x |  | x | x | E |
| CAPS-5** | x |  | x | x | E |
| DASS-21 | x |  | x | x | E |
| DERS-18 | x |  | x | x | E |
| Short SCS | x |  | x | x | E |
| IFF |  | x |  |  | A |
| IAQ |  |  | x |  | A |

GAIN-SS = Global Assessment of Individual Needs- Short Screen, MINI = Mini International Neuropsychiatric Interview, HSU = Health Service Utilization Questionnaire, GIAS = General Internet Attitude Scale, ACE = Adverse Childhood Experiences, PCL-5 = PTSD Checklist, CAPS-5 = Clinician-Administered PTSD Scale for DSM-5, DASS-21 = Depression and Anxiety Stress Scale, DERS-18 = Difficulties in Emotion Regulation Scale, Short SCS = Short Self Compassion Scale, IFF = Iterative Feedback Form, IAQ = Intervention Acceptability Questionnaire

R = recruitment, Co = covariates, E = efficacy, A = acceptability

*Includes sociodemographic and past medical and psychiatric history

**Completed by research staff

**2.7 Sample Size**

According to previous literature, a minimal clinically important difference between groups on the PCL would be 5-12 (median 7) points^28^. Our pilot study data allows us to estimate that the intervention group will go down by 12 points and the SD in the general population 15. Given that PCL-5 scores may change due to chance by up to 5 points, we estimate that the control group will go down by 5 points. At alpha 0.05 and 80% power (beta = 0.20), we will need N=73 per group for a total of 146 people.  Considering  potential for about 20% loss to follow-up, we will aim to recruit 200 people to ensure a primary outcome of 146.

Based on our pilot study, with approximately 25 participants enrolled in an Trauma PORTAL group at a time, the therapists were able to manage the asynchronous learning platform issues, and the “live” group was attended by a manageable number of participants each week (no more than about 8-10 participants attended any one group). Further, the follow-up rate in the pilot study was about 80% , such that with about 25 participants enrolled in a group, about 20 would be expected to complete the follow-up study measures.

If we aim to have approximately 25 participants in a cohort group at a time and run the full RCT protocol 4 times, this will result in a total sample size of 100 participants in the intervention group and 100 as controls. At 80% follow-up rates, this will mean a total of n=80/group. Given that we require n=73/group, this would be sufficient for power for our primary outcome even if follow-up rates are slightly below 80% or slightly fewer than 25 participants are enrolled in each group.

There are currently approximately 600 individuals on the current TTP waitlist, and there are expected to be about 100 new referrals per month. In the pilot study, approaching individuals on the TTP waitlist, we enrolled 6 per every 10 we screened for eligibility. As such, we expect our projected sample size is feasible and attainable as we recruit participants on a rolling basis as we fill up 8 cohorts of 25 participants over the course of 2 years.

**2.8 Recruitment, Retention, and Follow-Up**

**2.8.1 Recruitment and Follow-Up Procedures**

Study participants will be recruited from patients whose referrals to TTP have been accepted based on program eligibility criteria and who have attended the usual TTP orientation session. At the orientation session, potential participants will be provided with information about the study and asked to contact the research assistant if they are interested. They may also give their contact information via a link provided at the orientation session so they can be contacted by a research staff member. A member of the research team may attend orientation sessions to answer questions about the study. As an additional recruitment strategy, recruitment flyers explaining the study will be mailed out and sent via MyHealthRecord to individuals who have attended orientation. This may include individuals who have already been assessed for a “live video R&R” but remain on the waitling list for that intervention. Those who wish to participate may contact or be contacted by study research staff who will then explain the study, review eligibility criteria and conduct informed consent procedures. Participants will be told early in the recruitment process that, should they be assigned to the care-as-usual control group, participation in the study may still fast-track them to assessment by a TTP therapist compared to typical timelines, which is similar to the protocol for our eR&R Pilot Study.

**2.8.2 Retention Strategies**

To promote participant engagement with the Trauma PORTAL intervention RCT, we have included various interactive components in the Trauma PORTAL intervention, including skills-based distress tolerance exercises spaced throughout weekly modules and journaling prompts. As part of the study protocol, we have also established individual contact one of the Principal Investigators or a TTP therapist (via Zoom through MyHealth Record) for a clinical assessment to confirm eligibility. Furthermore, participants will receive a $60 gift card as a token of appreciation. $20 will be sent after completing the pre-intervention questionnaires, $20 will be sent upon completion of the post-intervention questionnaires, and the remaining $20 will be sent upon completion of the 16-week timepoint questionnaires. Weekly group facilitator providers have agreed to donate their time.

**2.8.3 Loss to Follow-Up**

We expect some attrition based on our team’s previous experience with a similar eHealth intervention (eR&R Pilot study and Mother Matters). Our aim to recruit 93 individuals per group will allow us to generate a sample size for this study after attrition. Using telephone and online follow-up with flexible timing and highly trained research personnel has resulted in follow-up rates of ~85% in previous RCTs by team members.

- 1. **Data Collection**

Research staff will conduct an initial eligibility assessment over the phone to confirm participants meet inclusion/exclusion criteria. If eligible, the research staff member will review the informed consent form with participants and will send the consent form to participants to review and sign if interested. Once consented, the research staff member will set up a virtual meeting between a Principal Investigator, both of whom are psychiatrists in the Trauma Therapy Program, or a TTP therapist, and participant for a 45-minute clinical assessment and to confirm that the participant is able to safely participate in the intervention and discuss group guidelines for the weekly online group component. This meeting will take place within three weeks of obtaining consent from participants. Once it is confirmed that the participant will be participating in the intervention, the baseline CAPS-5 assessment will be completed, and the participant will be enrolled in the trial.

The baseline assessment involves both a CAPS-5 telephone interview with a highly-trained research staff member and self-report measures completed by the participant via REDCap^TM^ (link sent to participant) (see timeline of outcome measures). The participant will then be randomized to the ITC or CUC group and the research staff member will assign them a unique study ID. The research staff member will then contact the participant to inform them of their group and start date and provide their login information to access the Trauma PORTAL platform if applicable.

Participants will be provided with an optional weekly feedback form via REDCap^TM^ to comment on the intervention, and potential areas for improvement, and then will complete post-intervention measures after the final week of the intervention (following the end of week 8). The participant will then exit the Trauma Portal Project study after which treatment group participants will be scheduled for a 45-minute standard clinical assessment visit with one of the Trauma Therapy Program therapists to discuss next steps for treatment as part of the standard treatment pathway. The results of the standard portion of this clinical assessment will be documented in the clinical chart, then extracted by chart review to be able to collect data on planned next steps for treatment after the intervention. All participants, in both control and treatment groups, will be assessed with the CAPS-5 for a second time by a research staff member.

Participants will then be asked to schedule a final phone call that will occur during their 16^th^ week of the trial. The research assistant will send out the secondary endpoint questionnaires at the 16-week mark. During this week, participants will have their final phone calls, where they will be assessed by the CAPS-5 interview for a final time.

Trauma PORTAL weekly group providers will be scheduled to complete a 30-minute debrief session with one of the Principal Investigators. Participants will be asked about their impressions of the group and main challenges. The debrief will be audio-recorded and transcribed for analysis.

**2.9.1 Data Management**

The secure encrypted web-based clinical trial data management system, REDCap^TM^ , will be used to conduct randomization and manage data collection. REDCap^TM^ is fully configurable and incorporates sophisticated data validation rules to ensure high quality data is captured for remote web-based data entered by participants, and research staff. WCH will host and manage the REDCap database.

WCH REDCap^TM^ administrators will create the web-based electronic case report forms (eCRFs). All data will be stored on local servers at WCH for the duration of the study and for up to 10 years after the study is complete. All study subjects will be identified in the database by a unique study ID number. Linkages between the participant name/contact information and the study ID will be retained by the research team and will not be shared with the WCH REDCap^TM^ administrators. At the end of the study, the REDCap administrators will transfer all study data to the PI. Data will only be accessible by authorized study personnel. Authorized personnel will receive a unique username and password to the REDCap website, and database access is controlled by the PI and the REDCap administrators.

All data collected in REDCap^TM^ is stored within the WCH network which features standard security protocols, and is encrypted with a 256-bit password. Backup of servers and the database occurs nightly. REDCap^TM^ is encrypted with SSL 1024-bit certificate and logs are automatically generated to track activity in every project.

The approving REB will be granted direct access to the study participants’ original study records for verification of trial procedures and/or data, without violating the confidentiality of the participants, to the extent permitted by law and regulations. In presenting results, participants’ identities will be confidential.

To minimize avoidable missing data in patient reported outcomes^26^, research staff will monitor submitted participant surveys for completion and follow up with participants via phone or email if there are any missing data.^27^ If the participant provides the missing information then research staff will report this to the delegated data manager who will enter the missing information into the participant's survey. Participants can choose to not answer questions. If a participant declines to answer a question, this will be documented as such.

Participant reported data that is not part of primary or secondary outcomes questionnaires including, but not limited to, dates, measurements, socio-demographic data and medical history will be queried for correctness, for example to ensure that correct dates and units of measure are reported. Research staff will follow-up with participants via phone or email to verify queried data. This will be documented as above.

1. **Analyses**

Proportions and means will be calculated to determine recruitment and retention outcomes including recruitment rate, rates of non-participation, and rates of follow-up, in addition to completion of baseline and post-intervention study measures, drop-out rate in each group at the primary endpoint (after final intervention week of the ITC group) and also report on any adverse events.

**3.1 Clinical measures**

A linear mixed-effects model for repeated measures will be used for the primary analysis to more accurately account for within-subject correlations over time. The ANCOVA is retained as a sensitivity analysis.. We will analyse the pre- and post-intervention efficacy scores for both the ITC and CUC as a secondary analysis.

We will conduct an on-treatment analysis of both clinical and scale and spread outcomes to understand the health system implications of those who adhere to the virtual program. We will do a sub-analysis of people who met the threshold score of 31 and up on PCL-5 and a separate sub-analysis of those who met the sub-clinical threshold (PCL score of 26-30).

**3.2 Health Service Scale and Spread Outcomes**

Proportions and means will be calculated to determine recruitment and retention outcomes including recruitment rate, rates of non-participation, and rates of follow-up. Acceptance outcomes will be calculated using Likert-type scale responses and comments from participant acceptability questionnaires. We will analyze whether Trauma PORTAL % completion and group participation determine health outcomes and subsequent steps participants took in TTP as determined in their exit interviews.

3.3 **Other Measures**

Responses on the HSU, GIAS, and ACE will be compared between the treatment group and control group to determine whether there are confounding/mediating/moderating variables in our findings. For example, does concurrent health service utilization confound any health benefits participants received during the time of treatment? Does attitude towards using the Internet moderate adherence to the treatment program?

**4.0 Trial Management**

**4.1 Coordination**

Day-to-day management will be coordinated by the experienced RA (under PI supervision and the Research Coordinator) who will obtain consent and collect all patient and provider data, including self-report questionnaires via REDCap^TM^. Study conduct will conform to the ICH Harmonised Tripartite Guideline for Good Clinical Practice. Data will be entered by staff authorized by the PI who will abide by confidentiality regulations of the Research Ethics Board (REB). All study forms will be checked regularly for completeness, internal consistency and consistency with other forms.

**4.2 Safety Monitoring**

Potential harms include transient worsening of psychiatric symptoms sometimes occurs during Stage I trauma treatment. We recognize that with the Trauma Portal, participants may find it more challenging to self-regulate without in-the-moment guidance of therapist given the asynchronous nature of the virtual platform. We will therefore integrate distress tolerance exercises into the weekly modules to break up the content and simulate pauses taken during in-person R&R to practice grounding techniques when one or more group members are dysregulated. Furthermore, we plan to minimize this potential harm by excluding participants with severe psychiatric needs (e.g. acute suicidality, active substance or alcohol use disorders, acute mania or psychosis), as well as having all participants undergo a clinical assessment by a Principal Investigator or TTP therapist to determine their readiness to complete the Trauma PORTAL intervention. In the Orientation Script, we advise participants to seek emergency medical services if they are at risk of imminent harm to self or others. Safety concerns will be managed clinically and on an ad-hoc basis as they come to the team’s attention. Participants will then be reassessed in terms of eligibility criteria (i.e. meeting stopping criteria) and suitability to continue with the Trauma PORTAL intervention.

A committee consisting of the principal and co-investigators and the research assistant will hold teleconferences as necessary to discuss study progress, including participant recruitment, and unexpected issues. Because of the low risk nature of this intervention, an independent Data Safety Monitoring Board will not be developed. Safety will be assessed at all time points and be routinely reviewed by the principal investigators. Adverse events will be recorded and serious adverse events immediately reported (within 24 hours by telephone or fax) to the WCH REB for consideration of further action (i.e. subject withdrawal, termination of study). Stopping criteria have been developed for this study with a focus on participant health and safety. If stopping criteria are met, the participant will be removed from the study and followed by their family physician. Participants who terminate early will be part of intention to treat analysis.

**4.3 Team**

***Dana Ross, MD, MSc, FRCPC*** (co-principal investigator) is a psychiatrist at the Trauma Therapy Program at WCH with extensive experience leading foundational stage I R&R groups, as well as later-stage trauma individual and group therapy. She organized and led the adaption of the in-person R&R materials into multimedia content on the Trauma PORTAL. Dr. Ross an Assistant Professor in the Department of Psychiatry at the University of Toronto (U of T). She is also the principal investigator of a project on the development and iterative optimization process of a virtual training module aimed at community healthcare professionals looking to deliver R&R groups in other communities. ***Nancy McCallum, MD, MSc, FRCPC*** (co-principal investigator) is the lead psychiatrist of the Trauma Therapy Program at WCH and an Assistant Professor in the Department of Psychiatry at the U of T. Dr. McCallum has been instrumental in the conception and development of the in-person R&R curriculum, as well as adapting these materials to be presented virtually on the Trauma PORTAL . ***Simone Vigod, MD, MSc, FRCPC (Co-investigator)*** is a psychiatrist and scientist at WCH and a Professor in the Department of Psychiatry at the U of T. Dr. Vigod has expertise in women’s mental health services research and RCTs. Dr. Vigod is the principal investigator of the pilot randomized waitlist control trial for Mother Matters, which is a similar eHealth intervention for postpartum mental health with a psychoeducational component and an asynchronous psychotherapeutic group component. ***Mahum Musheer, M.Ed., RP*** is a psychotherapist in the Trauma Therapy Program at WCH. She is an experienced trauma therapist who has been extensively involved in conceptualizing and developing stage 1 trauma therapy groups in TTP, including the in-person R&R curriculum and handbook. ***Sophie Soklaridis, PhD*,** is a Senior Scientist at CAMH and has extensive experience in the education scholarship field and has an excellent reputation as a prolific researcher in her field. Dr. Soklaridis is an associate professor in the Departments of Psychiatry and Family & Community Medicine in the Temerty FOM at the U of T. She has expertise in digital interventions to support mental health, and in rapid design thinking which encourages creative and innovative solutions and has a strong focus on the development of education scholarship initiatives that include issues such as equity, access, diversity, and social justice. ***David Rojas****,* ***PhD***, is a Scientistat the Wilson Centre. His program of research focuses on the evaluation of complex healthcare and educational systems. He is particularly interested in studying the construct of “emergence” or “unintended conseqences” due to the natural unfolding of educational-related systems. David is an advocate for bringing theory into program evaluation practices. As a Program Evaluation methodologist, he develops unique evaluation plans based on the characteristics of the system under evaluation to address the program’s complexity accurately. ***Tina Behdinan, MD, MSc*** is a resident in psychiatry at the University of Toronto. Under the supervision of Dr. Vigod, and with the assistance of the study research assistant, she will be responsible for study design and data analysis.

**5.0 Ethics**

**5.1 Research Ethics Approval**

Research ethics board approval will be sought at Women's College Hospital prior to the initiation of this trial. Ongoing approval will be obtained annually. Protocol amendments will not be implemented prior to approval by the REB, unless required to eliminate an immediate hazard, in which case the protocol deviation will be reported to the REB along with a protocol amendment.

**5.2 Informed Consent Process**

5.2.1 Patient Participants

Informed consent will be obtained before study assessments and procedures are performed and before any private information is recorded. A member of the research team will contact potential patient participants by phone to complete an eligibility assessment. Eligible patient participants will be provided with a clear explanation of the objectives, procedures, risks and benefits of the study and all questions will be answered. Questions will be asked of patient participants to ensure that they understand the nature of the research, risks and potential benefits of study participation, and their rights as research participants prior to signing the informed consent form. Patient participants will be emailed a copy of the informed consent form and asked to electronically sign the informed consent form and email it back to the research team before entry into the study. Potential patient participants will be allowed as much time as they would like to review the consent form prior to consent. After receiving the signed consent form from the patient participant, the research team member will send the form to the Principal Investigators for their signature, and once received, schedule an eligibility confirmation clinical assessment with a TTP therapist or one of the co-Principal Investigators.

**5.2.2 Provider Participants**

Consent from TTP therapist weekly group facilitators providers will be obtained via email. A member of the research team will email a copy of the consent form to the weekly group providers and ensure all questions are answered. Group provider participants will be asked to electronically sign the informed consent form and email it back to the research team before entry into the study. Potential participants will be allowed as much time as they would like to review the consent form prior to consent, but must consent before the group start date in order to participate.

**5.3 Confidentiality**

Eligibility screening data will be collected by the study research personnel by telephone and will be stored in the research study’s password-secured databases on the WCH server. For all other data collected at WCH, computer-based data will be entered into password-secured databases and paper files stored in a secure location. Data will only be accessible to study personnel.

The approving REB will be granted direct access to the study participants’ original study records for verification of trial procedures and/or data, without violating the confidentiality of the participants, to the extent permitted by law and regulations. In presenting results, participants’ identities will be confidential.

Computer based data will be entered into password-secured databases and paper files stored in a secure location. Data will only be accessible to study personnel. User profile & system data stored and transmitted by the study website is secured both technically and by business practices in compliance with both PHIPA and HIPPA standards. Data will be hosted entirely within Canada adhering to jurisdictional compliance standards. Data will be stored after the end of the study on a password protected electronic archive for the PI to access for 5 years. The approving REB will be granted direct access to the study participants’ original medical records for verification of trial procedures and/or data, without violating the confidentiality of the participants, to the extent permitted by law and regulations. In presenting results, participants’ identities will be confidential.

**6.0 Budget**

| **Item** | **Year 1** | **Year 2** |
| --- | --- | --- |
| *Personnel* | |  |
| WCH Research Assistant (0.6 FTE) | $32,600 | $34,100 |
| WCH Research Coordinator (0.4 FTE) | $29,500 | $30,900 |
| TOTAL PERSONNEL | $62,100 | $65,000 |
| *Materials and Supplies* | |  |
| Printing/photocopying | $300 | $300 |
| REDCap project development | $1,000 | $- |
| Mailers/postage | $150 | $150 |
| MINI License | $2,000 | $ - |
| Thinkific course platform | $ - | $1,600 |
| Participant tokens of appreciation $60 x 200 pts) | $6,000 | $6,000 |
| TOTAL MATERIALS AND EQUIPMENT | $9,450 | $8,050 |
| *Other* | |  |
| Conference Presentation | $ - | $1,000 |
| Open access manuscript publication | $ - | $3,000 |
| TOTAL OTHER | $ - | $4,000 |
| **TOTAL YEARLY COST** | **$** 71,550 | $77,050 |
| **TOTAL PROJECT COST** | **$148,600** | |

**7.0 References**

1. *World Report on Violence Against Children*. (2006).
2. Lanius, R., Vermetten, E. & Pain, C. *The impact of early life trauma on health and disease: The hidden epidemic*. (2010).
3. Mock, S. E. & Arai, S. M. Childhood Trauma and Chronic Illness in Adulthood: Mental Health and Socioeconomic Status as Explanatory Factors and Buffers. *Front. Psychol.* **1**, (2011).
4. Van Assche, L., Van de Ven, L., Vandenbulcke, M. & Luyten, P. Ghosts from the past? The association between childhood interpersonal trauma, attachment and anxiety and depression in late life. *Aging Ment. Health* 1–8 (2019) doi:10.1080/13607863.2019.1571017.
5. Gonzalez, A. *et al.* Childhood and family influences on depression, chronic physical conditions, and their comorbidity: Findings from the Ontario Child Health Study. *J. Psychiatr. Res.* **46**, 1475–1482 (2012).
6. Dugal, C. *et al.* Psychological Intimate Partner Violence and Childhood Cumulative Trauma: The Mediating Role of Affect Dysregulation, Maladaptive Personality Traits, and Negative Urgency. *J. Interpers. Violence* (2018) doi:10.1177/0886260518801022.
7. Bowlus, A., McKenna, K., Day, T. & Wright, D. The Economic Costs and Consequences of Child Abuse in Canada. 2003 Report to the Law Commission of Canada by Canadian Child Welfare Research Portal.
8. Cloitre, M. *et al.* The ISTSS Expert Consensus Treatment Guidelines for Complex PTSD in Adults. (2012).
9. Herman, J. L. *Trauma and Recovery: The Aftermath of Violence - From Domestic Abuse to Political Terror*. (Basic Books, 2015).
10. Van Ameringen, M., Turna, J., Khalesi, Z., Pullia, K. & Patterson, B. There is an app for that! The current state of mobile applications (apps) for DSM-5 obsessive-compulsive disorder, posttraumatic stress disorder, anxiety and mood disorders. *Depress. Anxiety* **34**, 526–539 (2017).
11. Paul, L. A., Hassija, C. M. & Clapp, J. D. Technological advances in the treatment of trauma: a review of promising practices. *Behav. Modif.* **36**, 897–923 (2012).
12. Christensen, H. *Increasing access and effectiveness: using the internet to deliver low intensity CBT*. (Oxford University Press, 2010).
13. Mohr, D. C. *et al.* Interest in behavioral and psychological treatments delivered face-to-face, by telephone, and by internet. *Ann. Behav. Med. Publ. Soc. Behav. Med.* **40**, 89–98 (2010).
14. Newman, M. G., Szkodny, L. E., Llera, S. J. & Przeworski, A. A review of technology-assisted self-help and minimal contact therapies for anxiety and depression: is human contact necessary for therapeutic efficacy? *Clin. Psychol. Rev.* **31**, 89–103 (2011).
15. Lewis, C., Roberts, N. P., Bethell, A., Robertson, L. & Bisson, J. I. Internet‐based cognitive and behavioural therapies for post‐traumatic stress disorder (PTSD) in adults. *Cochrane Database Syst. Rev.* **2018**, (2018).
16. Kuhn, E. *et al.* A randomized controlled trial of a smartphone app for posttraumatic stress disorder symptoms. *J. Consult. Clin. Psychol.* **85**, 267–273 (2017).
17. Yan, M. & Or, C. Factors in the 4-week Acceptance of a Computer-Based, Chronic Disease Self-Monitoring System in Patients with Type 2 Diabetes Mellitus and/or Hypertension. *Telemed. J. E-Health Off. J. Am. Telemed. Assoc.* **24**, 121–129 (2018).
18. Hirani, S. P. *et al.* Quantifying beliefs regarding telehealth: Development of the Whole Systems Demonstrator Service User Technology Acceptability Questionnaire. *J. Telemed. Telecare* **23**, 460–469 (2017).
19. Tasca, G. A. *et al.* The therapeutic factor inventory-8: Using item response theory to create a brief scale for continuous process monitoring for group psychotherapy. *Psychother. Res. J. Soc. Psychother. Res.* **26**, 131–145 (2016).
20. Maillet, É., Mathieu, L. & Sicotte, C. Modeling factors explaining the acceptance, actual use and satisfaction of nurses using an Electronic Patient Record in acute care settings: an extension of the UTAUT. *Int. J. Med. Inf.* **84**, 36–47 (2015).
21. Blevins, C. A., Weathers, F. W., Davis, M. T., Witte, T. K. & Domino, J. L. The Posttraumatic Stress Disorder Checklist for DSM-5 (PCL-5): Development and Initial Psychometric Evaluation. *J. Trauma. Stress* **28**, 489–498 (2015).
22. Ng, F. *et al.* The validity of the 21-item version of the Depression Anxiety Stress Scales as a routine clinical outcome measure. *Acta Neuropsychiatr.* **19**, 304–310 (2007).
23. Bjureberg, J. *et al.* Development and Validation of a Brief Version of the Difficulties in Emotion Regulation Scale: The DERS-16. *J. Psychopathol. Behav. Assess.* **38**, 284–296 (2016).
24. Joyce, M. & Kirakowski, J. Measuring Attitudes Towards the Internet: The General Internet Attitude Scale: International Journal of Human–Computer Interaction: Vol 31, No 8. *International Journal of Human–Computer Interaction* **31**,.
25. Hertzog, M. A. Considerations in determining sample size for pilot studies. *Res. Nurs. Health* **31**, 180–191 (2008).
26. Calvert M, King M, Mercieca-Bebber R, et al. SPIRIT-PRO Extension explanation and elaboration: guidelines for inclusion of patient-reported outcomes in protocols of clinical trials. *BMJ Open*. 2021;11:e045105.
27. Kyte D, Ives J, Draper H, et al. Current practices in patient-reported outcome (PRO) data collection in clinical trials: a cross-sectional survey of UK trial staff and management. *BMJ Open*. 2016;6:e012281.
28. Stefanovics, E. A., Rosenheck, R. A., Jones, K. M., Huang, G., & Krystal, J. H. Minimal Clinically Important Differences (MCID) in Assessing Outcomes of Post-Traumatic Stress Disorder. *The Psychiatric quarterly*, **89**(1), 141–155 (2018).
29. Felitti, V.J., Anda, R.F., Nordenberg, D, Williamson, D.F., Spitz A.M., Edwards, V.K., Koss, M.P., and Marks, J.S., (1998). Relationship of childhood abuse and household dysfunction to many of the leading causes of death in adults: The Adverse Childhood Experiences (ACE) Study. American Journal of Preventative Medicine, vol 14 (4), 245-258.
30. Neff, K.D. (2003). Development and validation of a scale to measure self-compassion. *Self and Identity,* 2, 223-250.
31. U.S. Department of Veterans Affairs. “Clinician-Administered PTSD Scale for DSM-5 (CAPS-5).” PTSD: National Center for PTSD. <https://www.ptsd.va.gov/professional/assessment/adult-int/caps.asp> Accessed 8 August 2022.
